# Supplementary material for: Hybrid chalcogen bonds in prodrug nanoassemblies provides dual redox-responsivity in the tumor microenvironment
Source: Nat Commun. 2022 Nov 24;13:7228. doi: 10.1038/s41467-022-35033-7 (PMC9700694; doi:10.1038/s41467-022-35033-7)
Supplement: Supplementary file 1 — Supplementary Information [file 41467_2022_35033_MOESM1_ESM.pdf]

Supplementary Information

Hybrid Chalcogen Bonds in Prodrug Nanoassemblies Provides Dual Redox-Responsivity in the Tumour Microenvironment

Liu et al.

Supplementary Figures

a

b

c

d

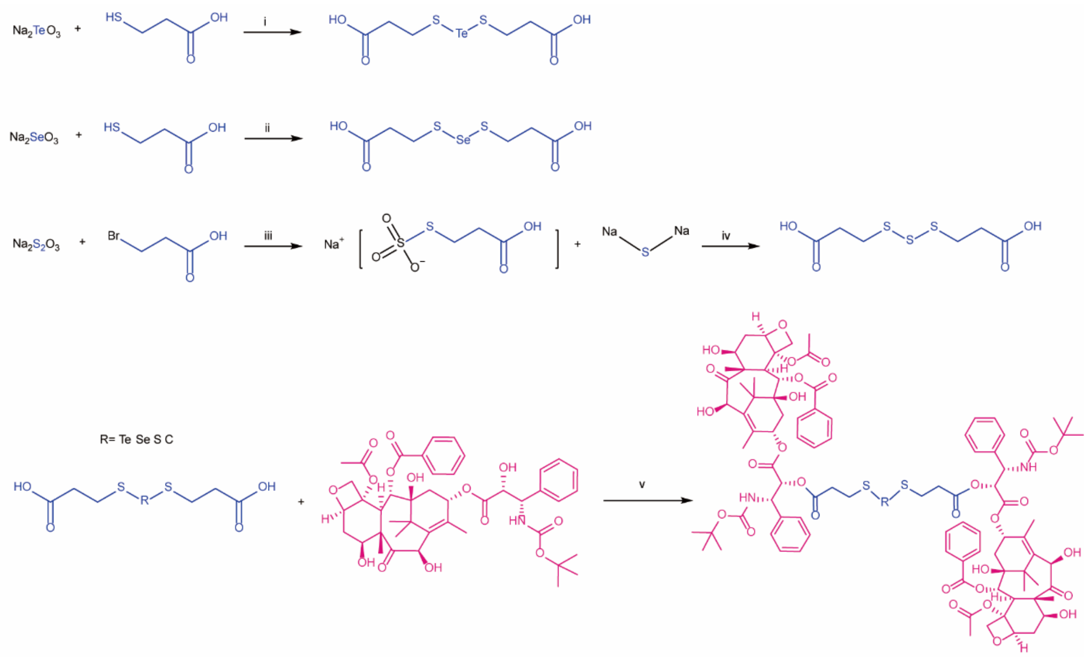

Supplementary Figure 1. Synthetic routes of prodrugs. **a** The synthetic of 3,3'-(telluriumdithio)-dipropionic acid **b** The synthetic of 3,3'-(selenodithio)-dipropionic acid. **c** The synthetic of 3,3'-trithiodipropionic acid. **d** The synthetic of DTX homodimeric prodrugs. Reagents and condition: (i) H_2O, 25°C; (ii) 0.5 mol L^{-1} H_2SO_4, 25°C; (iii) H_2O, 50-60°C; (iv) H_2O, 25°C; (v) EDCI, DMAP, $CHCl_3$, 25°C.

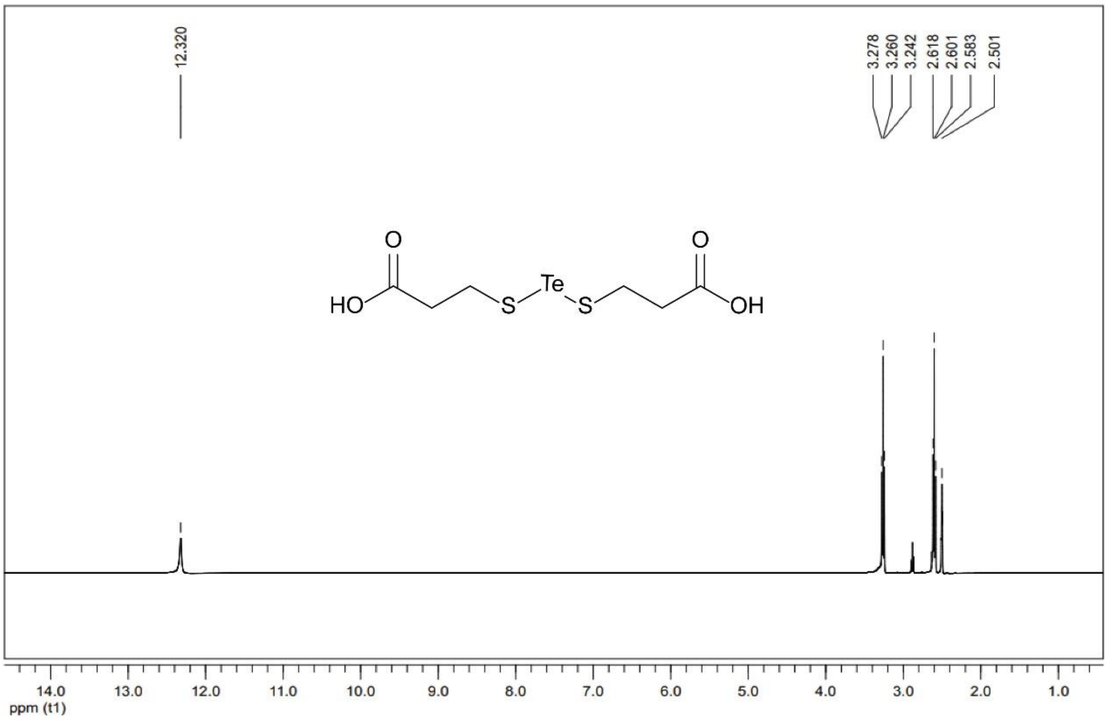

Supplementary Figure 2. ^{1}H NMR spectrum of 3,3'-(telluriumdithio)-dipropionic acid. ^{1}H NMR (400 MHz, DMSO-d6) δ ppm 12.32 (s, 2H, -COOH), 3.26 (t, J = 7.11 Hz, 4H, $COOHCH_2\underline{CH_2}STeS\underline{CH_2}CH_2COOH$), 2.60 (t, J = 7.11 Hz, 4H, $COOH\underline{CH_2}CH_2STeS$ $CH_2\underline{CH_2}COOH$).

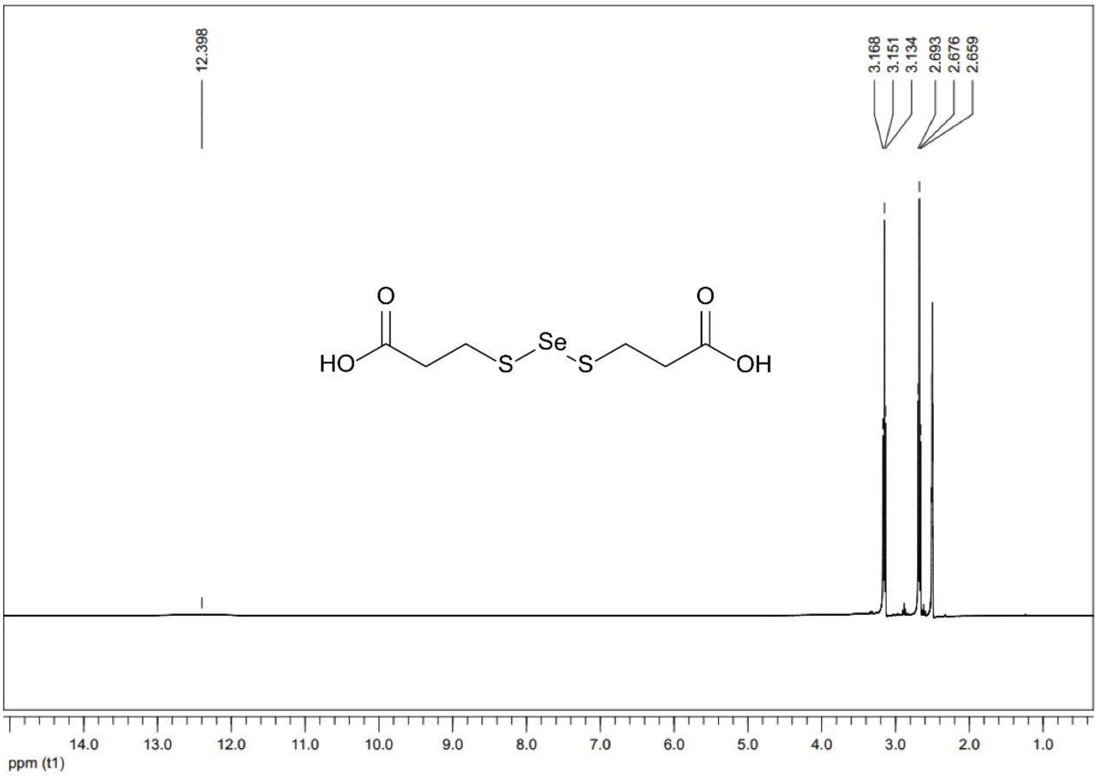

Supplementary Figure 3. ^{1}H NMR spectrum of 3,3'-(selenodithio)-dipropionic acid. ^{1}H NMR (400 MHz, DMSO-d6) δ ppm 12.63-12.13 (m, 2H, -COOH), 3.15 (t, J = 6.97 Hz, 4H, $COOHCH_2\underline{CH_2}SSeS\underline{CH_2}CH_2COOH$), 2.68 (t, J = 6.96 Hz, 4H, $COOH\underline{CH_2}CH_2SSeS\ CH_2\underline{CH_2}COOH$).

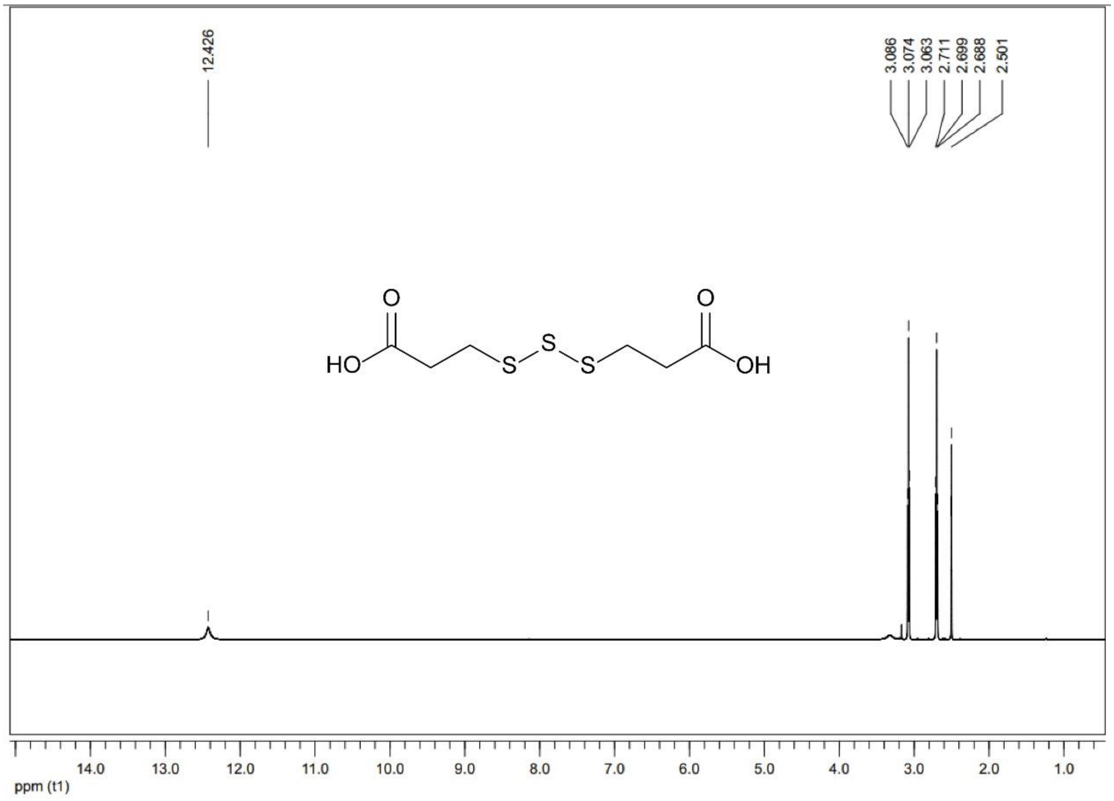

Supplementary Figure 4. ^{1}H NMR spectrum of 3,3'-trithiodipropionic acid.

^{1}H NMR (600 MHz, DMSO-d6) δ ppm 12.43 (s, -COOH, 2H), 3.07 (t, J = 6.92 Hz, 4H, $COOHCH_2\underline{CH_2}SSS\underline{CH_2}CH_2COOH$), 2.70 (t, J = 6.92 Hz, 4H, $COOH\underline{CH_2}CH_2SSS$ $CH_2\underline{CH_2}COOH$).

a

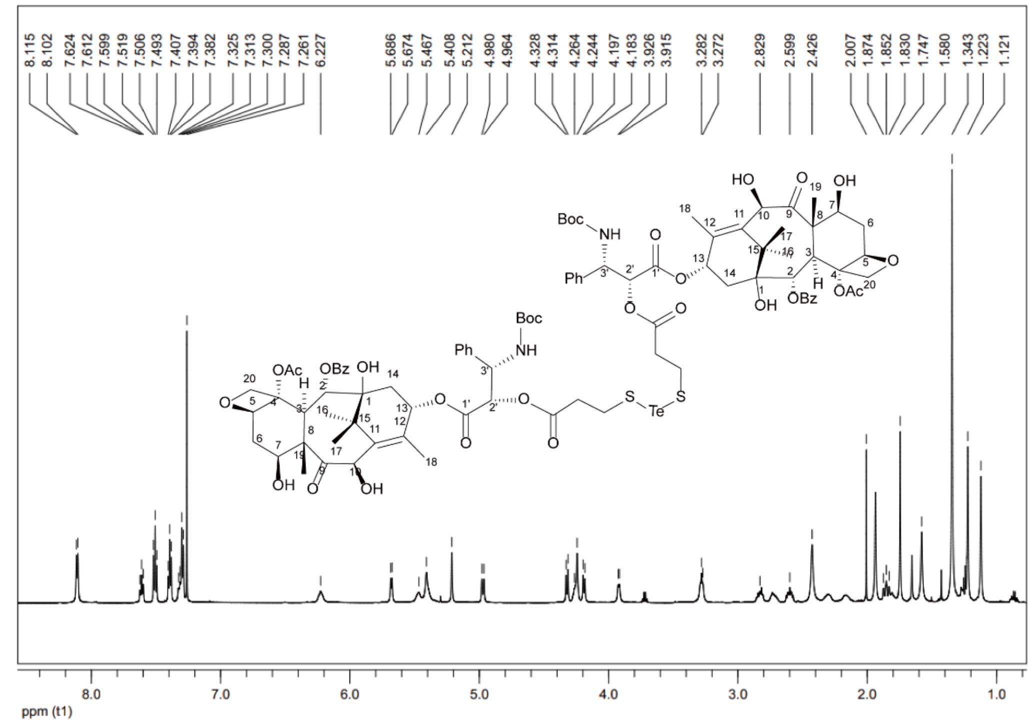

b

Acquisition Parameter

Acquisition Mode	Single MS	Acquired Scans	2	Calibration Date	Wed Nov 6 09:51:51 2019
Polarity	Positive	No. of Cell Fills	1	Data Acquisition Size	1048576
Broadband Low Mass	100.3 m/z	No. of Laser Shots	500	Data Processing Size (SI)	2097152
Broadband High Mass	2000.0 m/z	Laser Power	20.0 lp	Apodization	Full-Sine
Source Accumulation	0.000 sec	Laser Shot Frequency	0.001 sec		
Ion Accumulation Time	0.280 sec				

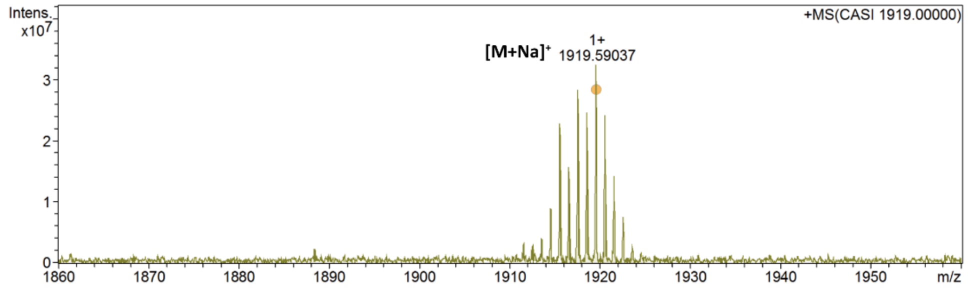

Meas. m/z	#	Ion Formula	Score	m/z	err [ppm]
1919.590374	1	C92H113N2O30S2Te	100.00	1919.587633	-0.7

c

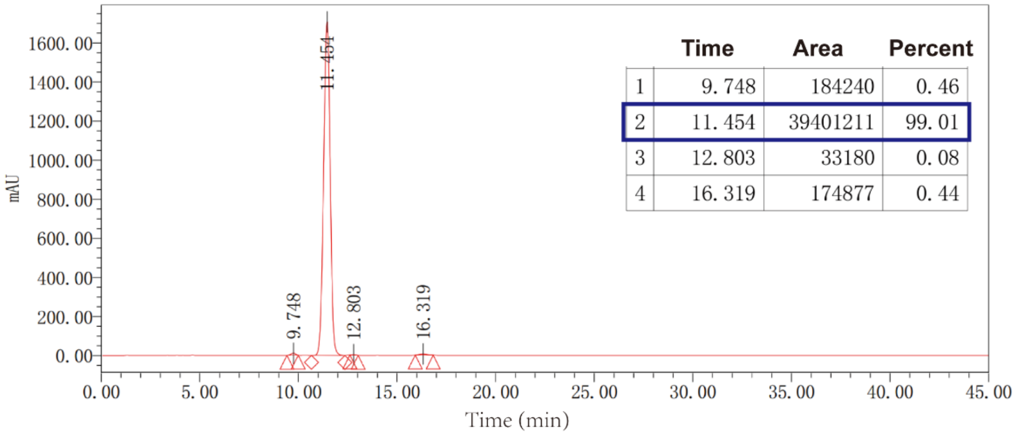

	Time	Area	Percent
1	9. 748	184240	0. 46
2	11. 454	39401211	99. 01
3	12. 803	33180	0. 08
4	16. 319	174877	0. 44

Supplementary Figure 5. Characterizations of DTX-STeS-DTX. **a** ^{1}H NMR spectrum. **b** Mass spectrum. **c** The purity.

^{1}H NMR (600 MHz, CDCl3) δ ppm 8.11 (d, J = 7.61 Hz, 4H, Ar-H), 7.61 (t, J = 7.37 Hz, 2H, Ar-H), 7.51 (t, J = 7.72 Hz, 4H, Ar-H), 7.39 (t, J = 7.53 Hz, 4H, Ar-H), 7.31 (dd, J = 15.15, 7.34 Hz, 6H, Ar-H), 6.23 (s, 2H, 13-CH), 5.68 (d, J = 6.90 Hz, 2H, 2-CH), 5.47 (s, 2H, 3'-CH), 5.41 (s, 2H, 10-CH), 5.21 (s, 2H, 2'-CH), 4.97 (d, J = 9.21 Hz, 2H , 5-CH), 4.32 (d, J = 8.55 Hz, 2H, 20-CH_2-αH), 4.25 (d, J = 12.02 Hz, 2H, 7-CH), 4.19 (d, J = 8.48 Hz, 2H, 20-CH_2-βH), 3.92 (d, J = 6.31 Hz, 2H, 3-CH), 3.28 (s, 4H, $CH_2\underline{CH_2}STeS\underline{CH_2}CH_2$), 2.86-2.79 (m, 2H, $\underline{CH_2}CH_2STeSCH_2\underline{CH_2}$-αH), 2.63-2.56 (m, 2H, $\underline{CH_2}CH_2STeSCH_2\underline{CH_2}$-βH), 2.43 (s, 6H, -OAc), 2.01 (s, 2H, 6-CH_2-αH), 1.88-1.83 (m, 2H, 6-CH_2-βH), 1.75 (s, 6H, 19-CH_3), 1.58 (s, 4H, 14-CH_2), 1.34 (s, 18H, -Boc), 1.22 (s, 6H, 16-CH_3), 1.12 (s, 6H, 17-CH_3).

HRMS calcd. for $C_{92}H_{112}N_2O_{30}S_2Te$, (ESI) m/z $[M+Na]^+$ = 1919.59037.

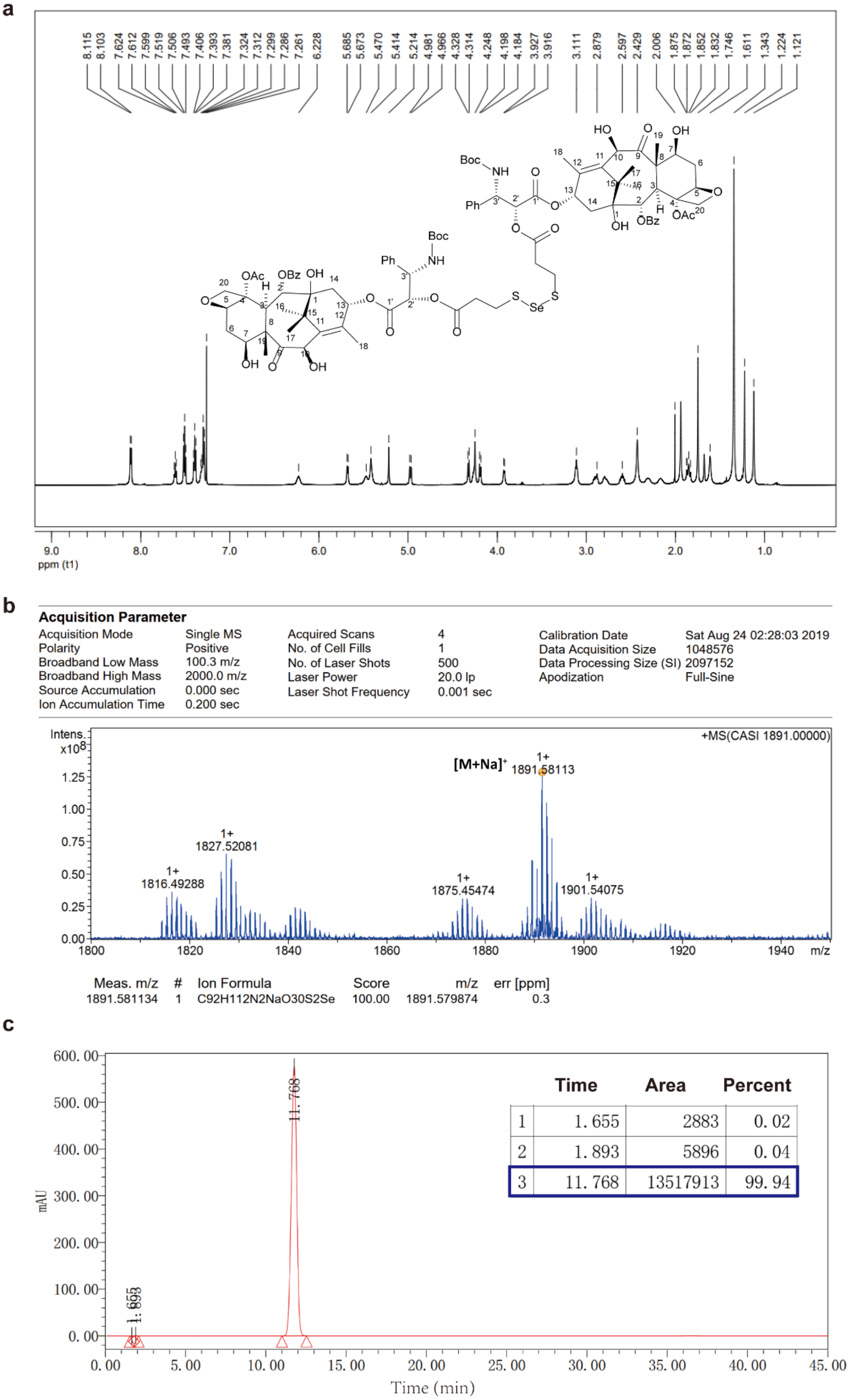

Supplementary Figure 6. Characterizations of DTX-SSeS-DTX. **a** ^{1}H NMR spectrum. **b** Mass spectrum. **c** The purity.

^{1}H NMR (600 MHz, $CDCl_3$) δ ppm 8.109 (d, J = 7.55 Hz, 4H, Ar-H), 7.612 (t, J = 7.30 Hz, 2H, Ar-H), 7.506 (t, J = 7.74 Hz, 4H, Ar-H), 7.393 (t, J = 7.54 Hz, 4H, Ar-H), 7.305 (dd, J = 15.57, 7.36 Hz, 6H, Ar-H), 6.228 (s, 2H, 13-CH), 5.679 (d, J = 6.82 Hz, 2H, 2-CH), 5.470 (s, 2H, 3'-CH), 5.414 (s, 2H, 10-CH), 5.214 (s, 2H, 2'-CH), 4.974 (d, J = 9.21 Hz, 2H, 5-CH), 4.321 (d, J = 8.53 Hz, 2H, 20-CH_2-αH), 4.248 (s, 2H, 7-CH), 4.191 (d, J = 8.51 Hz, 2H, 20-CH_2-βH), 3.921 (d, J = 6.11 Hz, 2H, 3-CH), 3.111 (s, 4H, $CH_2\underline{CH_2}SSeS\underline{CH_2}CH_2$), 2.934-2.856 (m, 2H, $\underline{CH_2}CH_2SSeSCH_2\underline{CH_2}$-αH), 2.643-2.564 (m, 2H, $\underline{CH_2}CH_2SSeSCH_2\underline{CH_2}$-βH), 2.429 (s, 6H, -OAc), 2.006 (s, 2H, 6-CH_2-αH), 1.858 (dd, 2H, 6-CH_2-βH), 1.746 (s, 6H, 19-CH_3), 1.611 (s, 4H, 14-CH_2), 1.343 (s, 18H, -Boc), 1.232 (d, 6H, 16-CH_3), 1.121 (s, 6H, 17-CH_3)

HRMS calcd. for $C_{92}H_{112}N_2O_{30}S_2Se$, (ESI) m/z $[M+Na]^+$ =1891.58113.

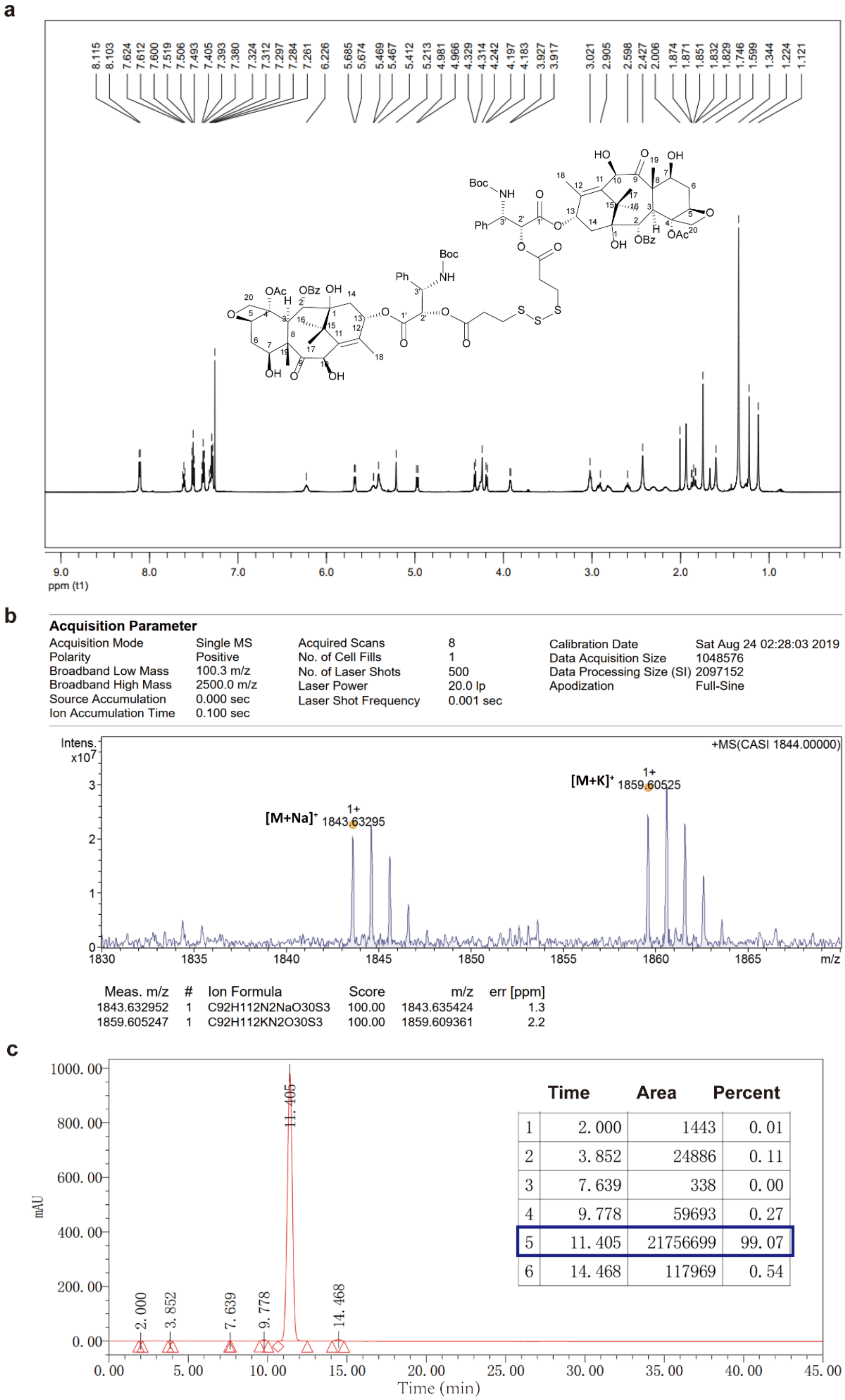

Supplementary Figure 7. Characterizations of DTX-SSS-DTX. **a** ^{1}H NMR spectrum. **b** Mass spectrum. **c** The purity.

^{1}H NMR (600 MHz, $CDCl_3$) δ ppm 8.109 (d, J = 7.59 Hz, 4H, Ar-H), 7.612 (t, J = 7.36 Hz, 2H, Ar-H), 7.506 (t, J = 7.74 Hz, 4H, Ar-H), 7.393 (t, J = 7.56 Hz, 4H, Ar-H), 7.304 (dd, J = 16.65, 7.32 Hz, 6H, Ar-H), 6.226 (s, 2H, 13-CH), 5.680 (d, J = 6.90 Hz, 2H, 2-CH), 5.468 (d, J = 0.90 Hz, 2H, 3'-CH), 5.412 (s, 2H, 10-CH), 5.213 (s, 2H, 2'-CH), 4.973 (d, J = 9.01 Hz, 2H, 5-CH), 4.322 (d, J = 8.54 Hz, 2H, 20-CH_2-αH), 4.285-4.222 (m, 2H, 7-CH), 4.190 (d, J = 8.51 Hz, 2H, 20-CH_2-βH), 3.922 (d, J = 6.33 Hz, 2H, 3-CH), 3.016 (d, 4H, $CH_2\underline{CH_2}SSS\underline{CH_2}CH_2$), 2.919 (td, 2H, $\underline{CH_2}CH_2SSSCH_2\underline{CH_2}$-αH), 2.636-2.566 (m, 2H, $\underline{CH_2}CH_2SSSCH_2\underline{CH_2}$-βH), 2.427 (s, 6H, -OAc), 2.006 (s, 2H, 6-CH_2-αH), 1.884-1.816 (m, 2H, 6-CH_2-βH), 1.746 (s, 6H, 19-CH_3), 1.599 (s, 4H, 14-CH_2), 1.344 (s, 18H, -Boc), 1.253-1.204 (m, 6H, 16-CH_3), 1.121 (s, 6H, 17-CH_3)

HRMS calcd. for $C_{92}H_{112}N_2O_{30}S_3$, (ESI) m/z $[M+Na]^+$ =1843.63295, $[M+K]^+$ = 1859.60525.

a

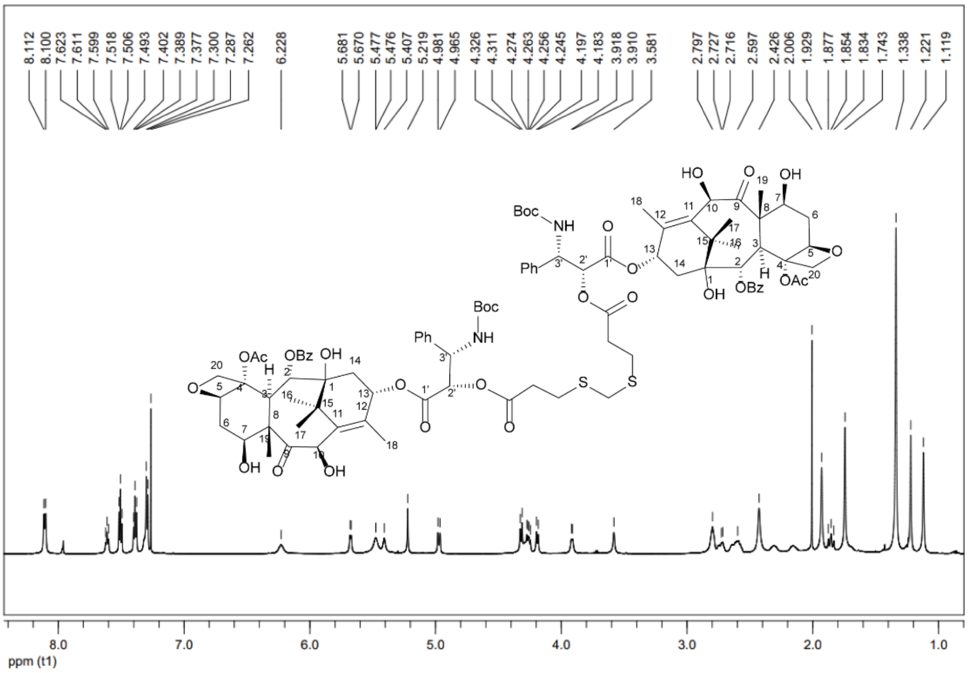

b

Acquisition Parameter

Acquisition Mode	Single MS	Acquired Scans	8	Calibration Date	Thu Dec 12 09:38:20 2019
Polarity	Positive	No. of Cell Fills	1	Data Acquisition Size	1048576
Broadband Low Mass	100.3 m/z	No. of Laser Shots	500	Data Processing Size (SI)	2097152
Broadband High Mass	2000.0 m/z	Laser Power	20.0 lp	Apodization	Full-Sine
Source Accumulation	0.000 sec	Laser Shot Frequency	0.001 sec		
Ion Accumulation Time	0.200 sec				

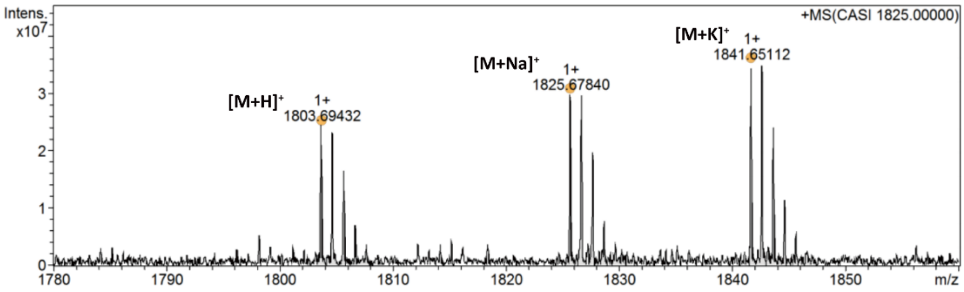

Meas. m/z	#	Ion Formula	Score	m/z	err [ppm]
1803.694323	1	C93H115N2O30S2	100.00	1803.697059	1.5
1825.678397	1	C93H114N2NaO30S2	100.00	1825.679003	0.3
1841.651116	1	C93H114KN2O30S2	100.00	1841.652940	1.0

c

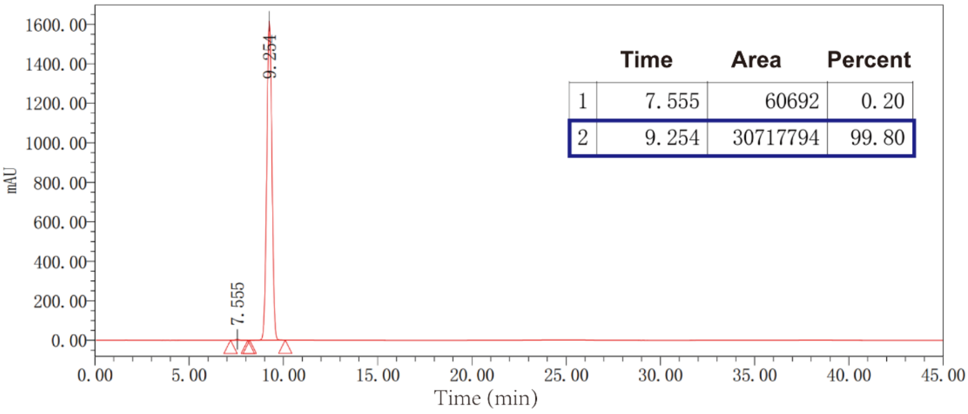

Supplementary Figure 8. Characterizations of DTX-SCS-DTX. **a** ^{1}H NMR spectrum. **b** Mass spectrum. **c** The purity.

^1H NMR (600 MHz, $CDCl_3$) δ ppm 8.106 (d, J = 7.49 Hz, 4H, Ar-H), 7.611 (t, J = 7.18 Hz, 2H, Ar-H), 7.506 (t, J = 7.68 Hz, 4H, Ar-H), 7.389 (t, J = 7.54 Hz, 4H, Ar-H), 7.293 (d, J = 8.00 Hz, 6H, Ar-H), 6.228 (s, 2H, 13-CH), 5.675 (d, J = 6.37 Hz, 2H, 2-CH), 5.477 (d, J = 0.68 Hz, 2H), 5.407 (s, 2H, 10-CH), 5.219 (s, 2H, 2'-CH), 4.973 (d, J = 9.36 Hz, 2H, 5-CH), 4.319 (d, J = 8.55 Hz, 2H, 20-CH_2-αH), 4.260 (dd, J = 10.84, 6.74 Hz, 2H, 7-CH), 4.190 (d, J = 8.43 Hz, 2H, 20-CH_2-βH), 3.914 (d, J = 4.94 Hz, 2H, 3-CH), 3.581 (s, 4H, $CH_2\underline{CH_2}SCH_2S\underline{CH_2}CH_2$), 2.797 (s, 2H, $CH_2CH_2S\underline{CH_2}SCH_2CH_2$), 2.797-2.716 (m, 2H, $\underline{CH_2}CH_2SCH_2SCH_2\underline{CH_2}$-αH) 2.619-2.564 (m, 2H, $\underline{CH_2}CH_2SCH_2SCH_2\underline{CH_2}$-βH), 2.426 (s, 6H, -OAc), 2.001 (d, J = 5.26 Hz, 2H, 6-CH_2-αH), 1.929 (s, 4H, 14-CH_2), 1.885-1.823 (m, 2H, 6-CH_2-βH), 1.743 (s, 6H, 19-CH_3), 1.338 (s, 18H, -Boc), 1.221 (s, 6H, 16-CH_3), 1.119 (s, 6H, 17-CH_3)

HRMS calcd. for $C_{93}H_{114}N_2O_{30}S_2$, (ESI) m/z $[M+H]^+$ = 1803.69432, $[M+Na]^+$ = 1825.67840, $[M+K]^+$ = 1841.65112.

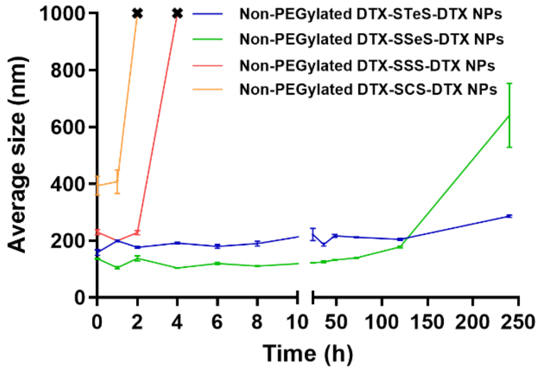

Supplementary Figure 9. Non-PEGylated HPNAs stored at room temperature for 240 h. Data are presented as mean ± SD (n = 3 independent experiments). Source data are provided as a Source Data file.

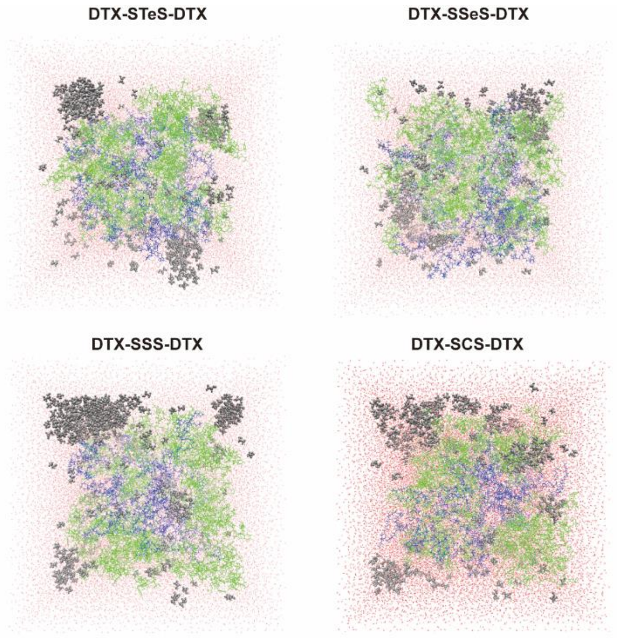

Supplementary Figure 10. The initial conformation of the system. The red represents water, the gray structure represents ethanol, the blue structure represents DSPE-PEG$_{2K}$, and the green structure represents prodrugs.

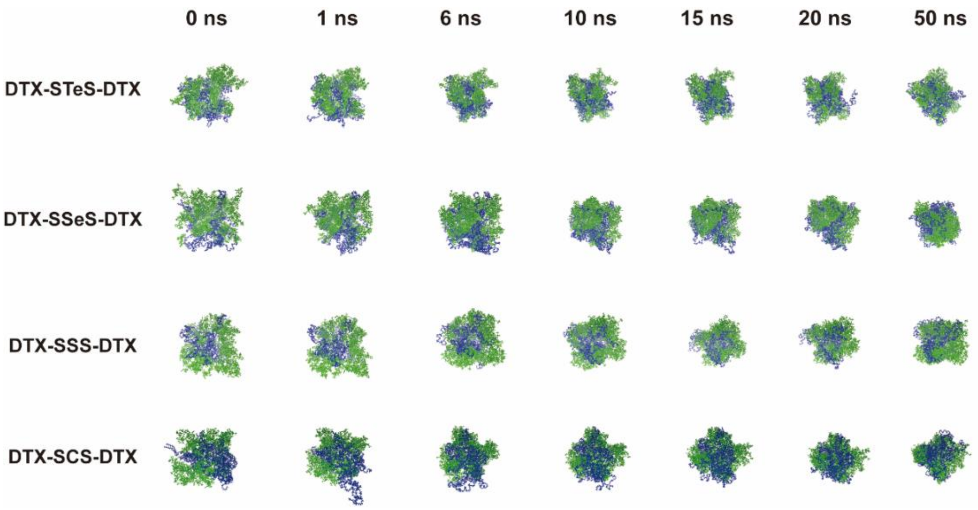

Supplementary Figure 11. Track of self-assembly conformation of prodrug in the system with time. The blue structure represents DSPE-PEG$_{2K}$, and the green structure represents prodrugs.

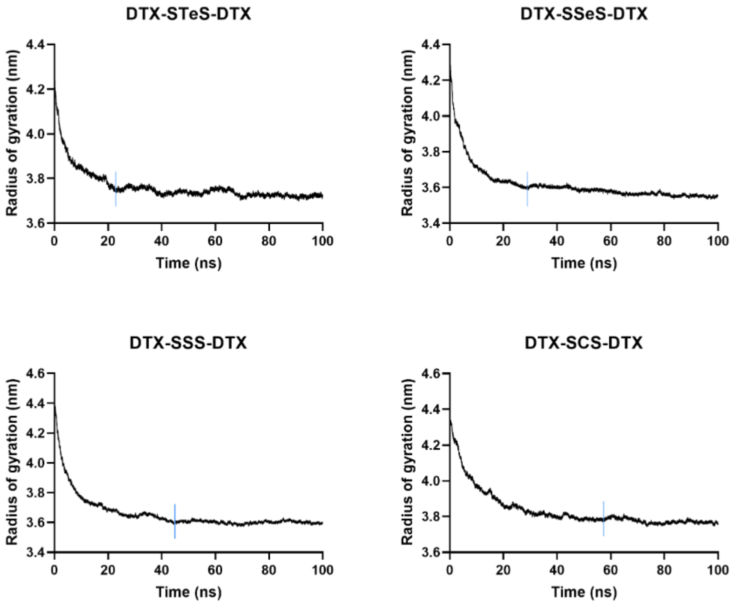

Supplementary Figure 12. The radius of gyration of prodrug and DSPE-PEG_{2K} during aggregation. Source data are provided as a Source Data file.

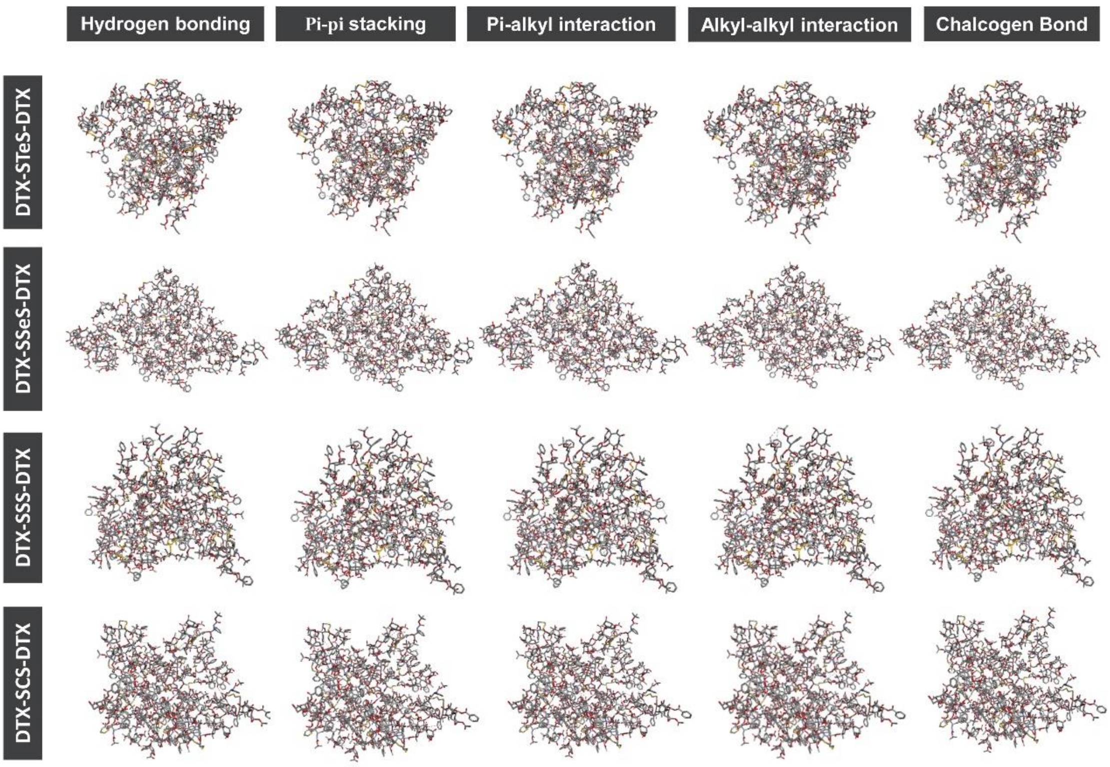

Supplementary Figure 13. Noncovalent interactions in determining the conformational stability of HPNAs.

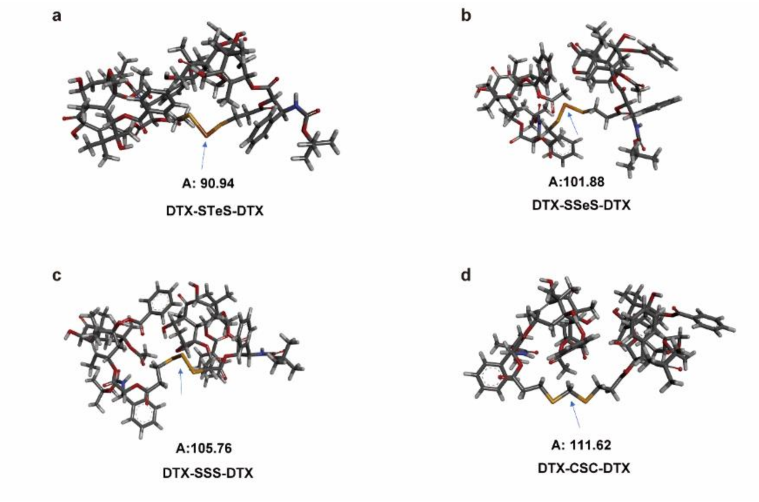

Supplementary Figure 14. The optimized geometry structure and bond angles of prodrugs. **a** DTX-S<u>Te</u>S-DTX, **b** DTX-S<u>Se</u>S-DTX, **c** DTX-S<u>S</u>S-DTX, and **d** DTX-S<u>C</u>S-DTX.

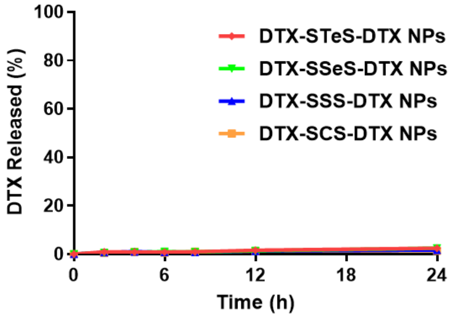

Supplementary Figure 15. In vitro drug release of HPNAs in the medium without redox substance. Data are presented as mean ± SD (n = 3 independent experiments). Source data are provided as a Source Data file.

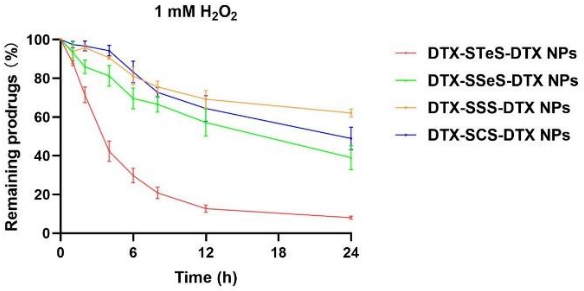

Supplementary Figure 16. In vitro the response rate of HPNAs under 1 mM H_2O_2. Data are presented as mean ± SD (n = 3 independent experiments). Source data are provided as a Source Data file.

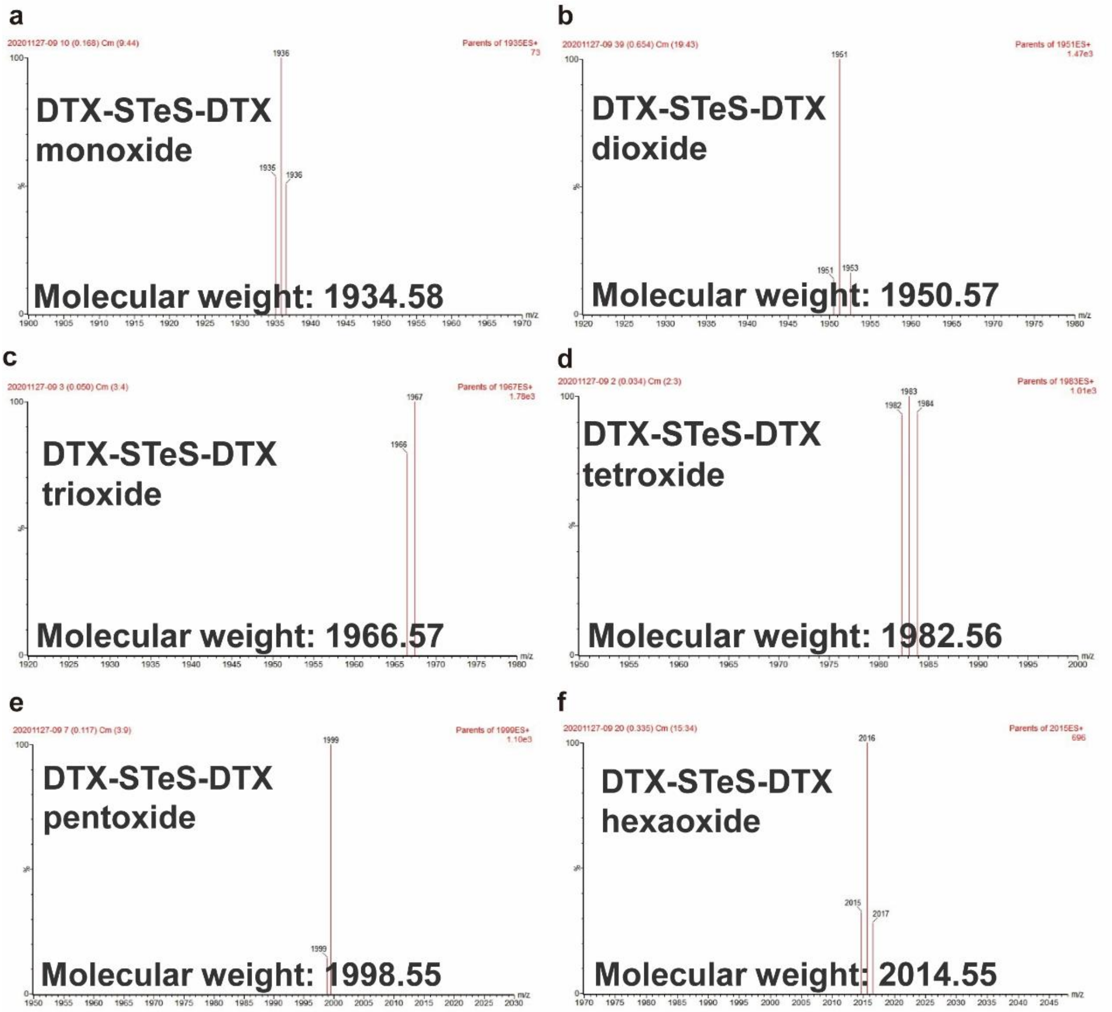

Supplementary Figure 17. In vitro oxidation-responsive release intermediate of DTX-STeS-DTX. **a**-**f** Mass spectra of mono-to hexaoxide of DTX-STeS-DTX after incubated with H_2O_2-containing release media.

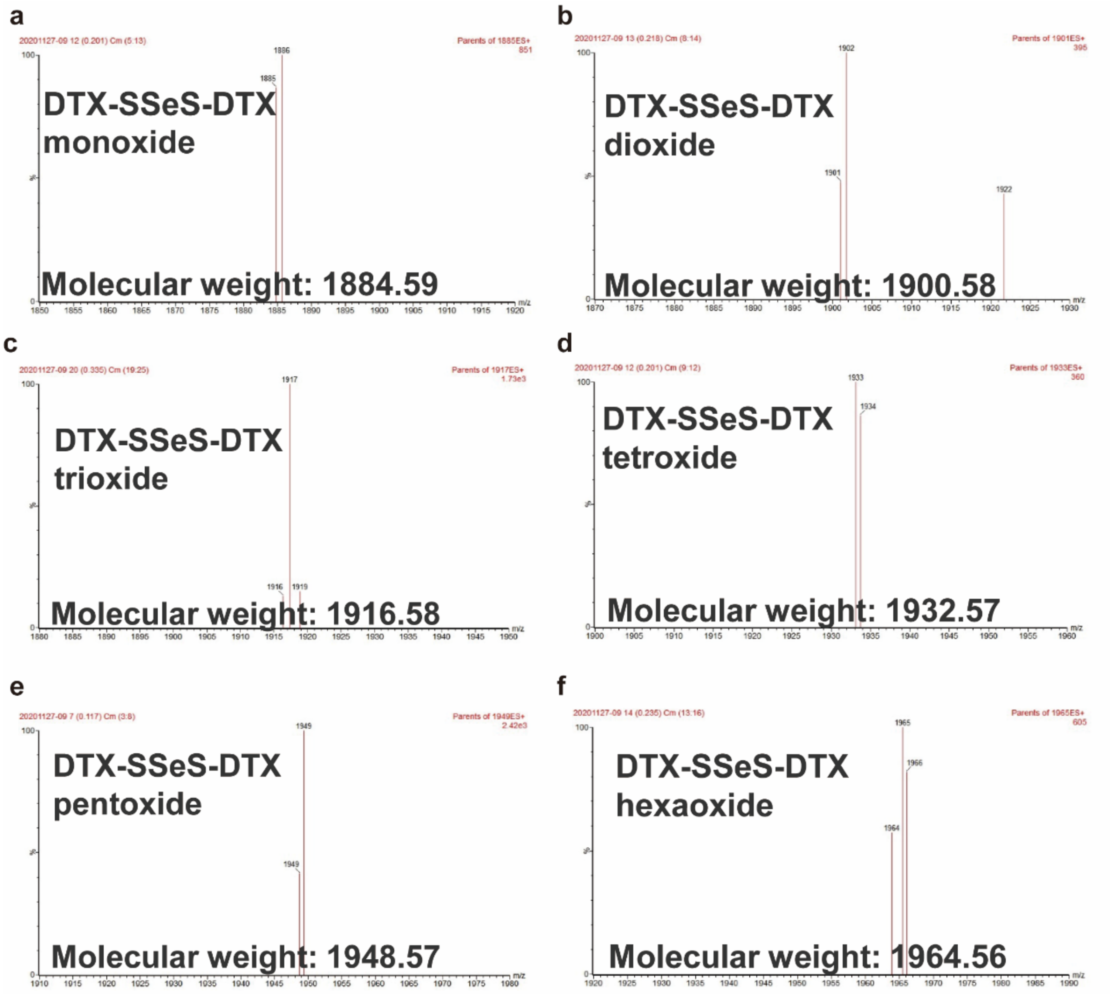

Supplementary Figure 18. In vitro oxidation-responsive release intermediate of DTX-SSeS-DTX. **a-f** Mass spectra of mono-to hexaoxide of DTX-SSeS-DTX after incubated with H_2O_2-containing release media.

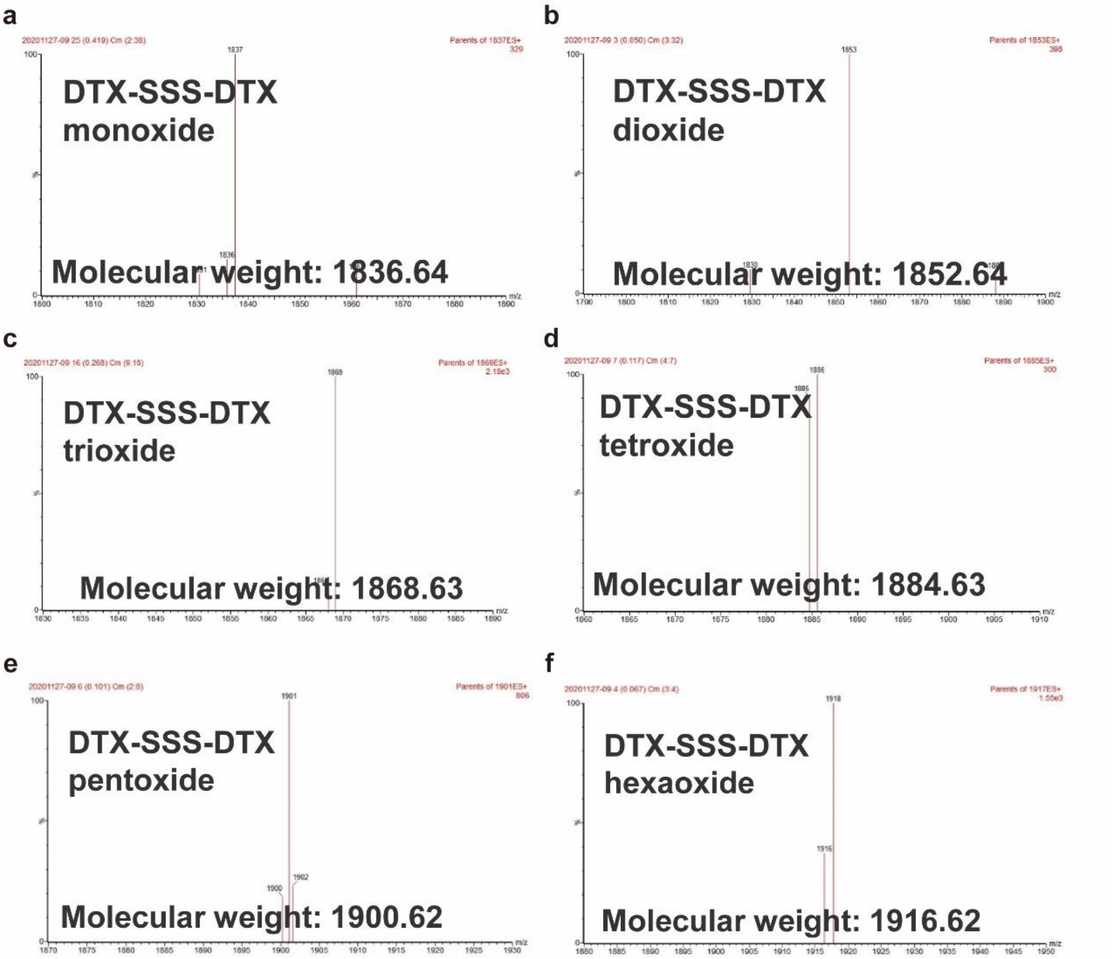

Supplementary Figure 19. In vitro oxidation-responsive release intermediate of DTX-SSS-DTX. **a**-**f** Mass spectra of mono-to hexaoxide of DTX-SSS-DTX after incubated with H_2O_2-containing release media.

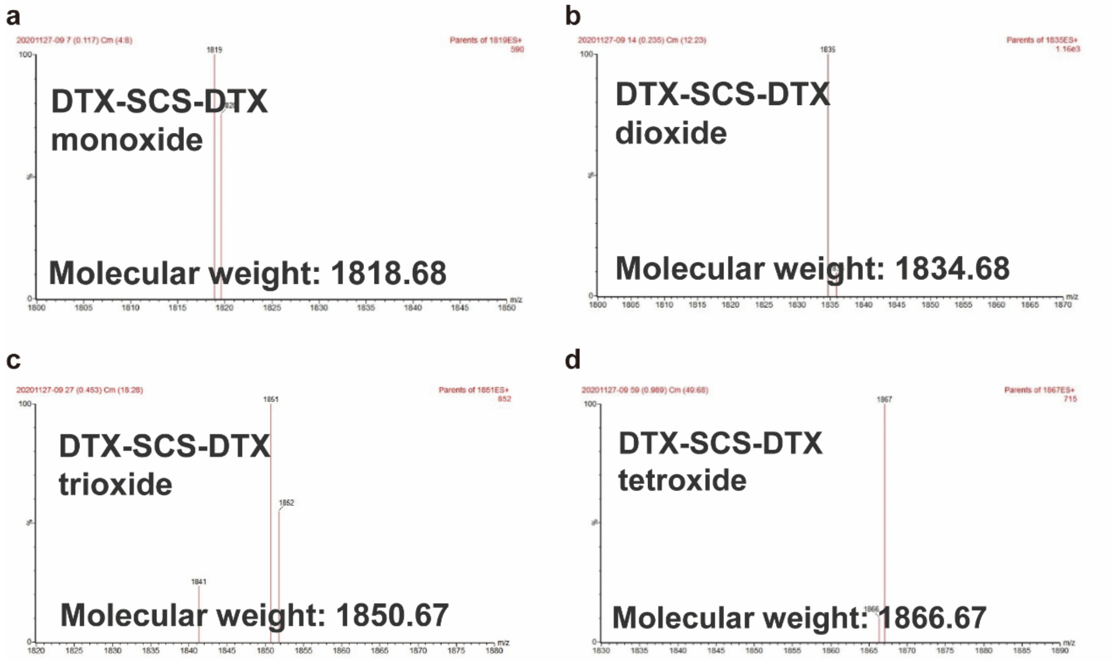

Supplementary Figure 20. In vitro oxidation-responsive release intermediate of DTX-SCS-DTX. **a-d** Mass spectra of mono-to tetroxide of DTX-SCS-DTX after incubated with H_2O_2-containing release media.

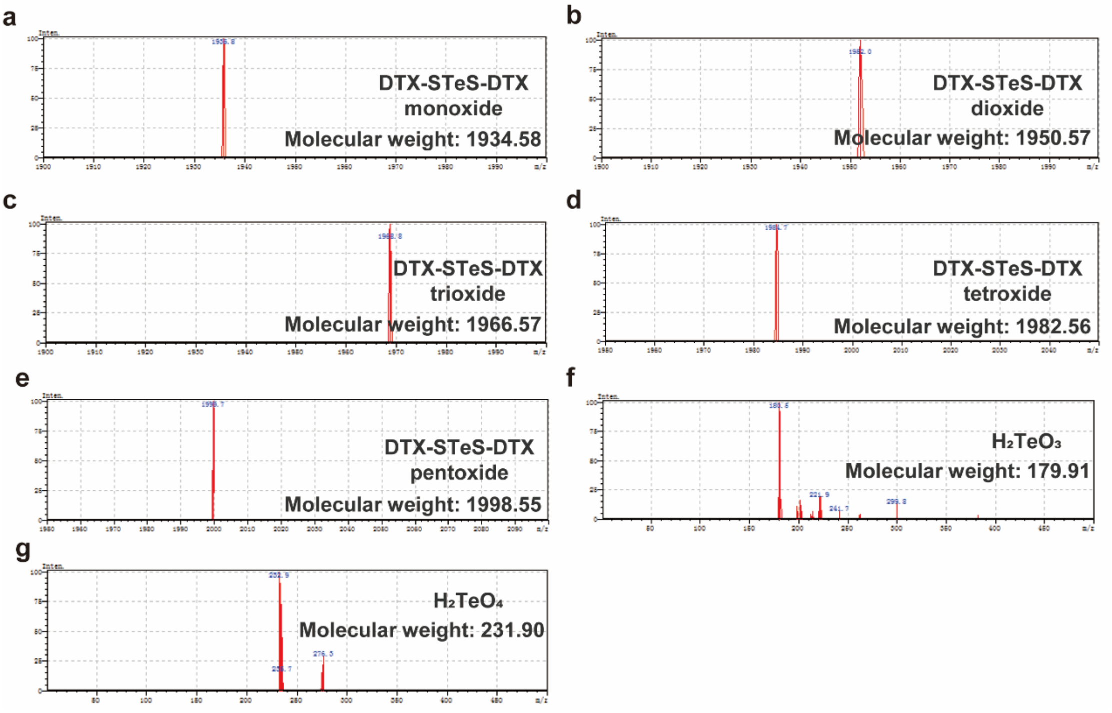

Supplementary Figure 21. Mass spectra of intracellular oxidation-responsive intermediates of DTX-STeS-DTX NPs. **a-e** monoxide to pentoxide of DTX-STeS-DTX. **f-g** tellurite acid and telluric acid.

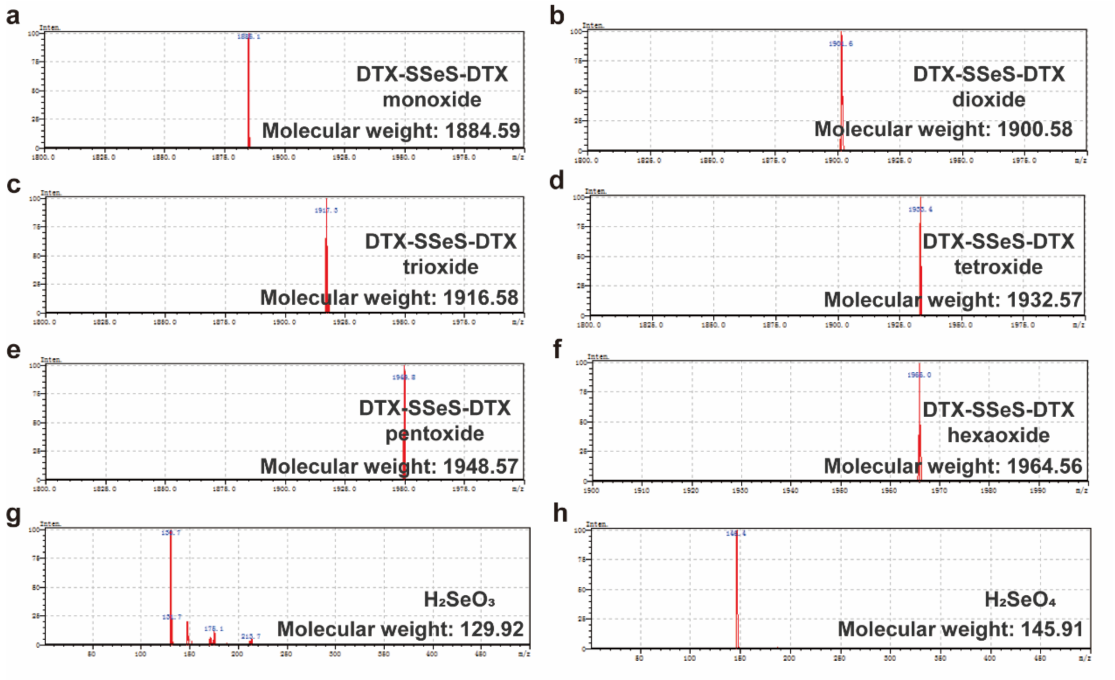

Supplementary Figure 22. Mass spectra of intracellular oxidation-responsive intermediates of DTX-SSeS-DTX NPs. **a-f** monoxide to hexaoxide of DTX-SSeS-DTX. **g-h** selenous acid and selenic acid.

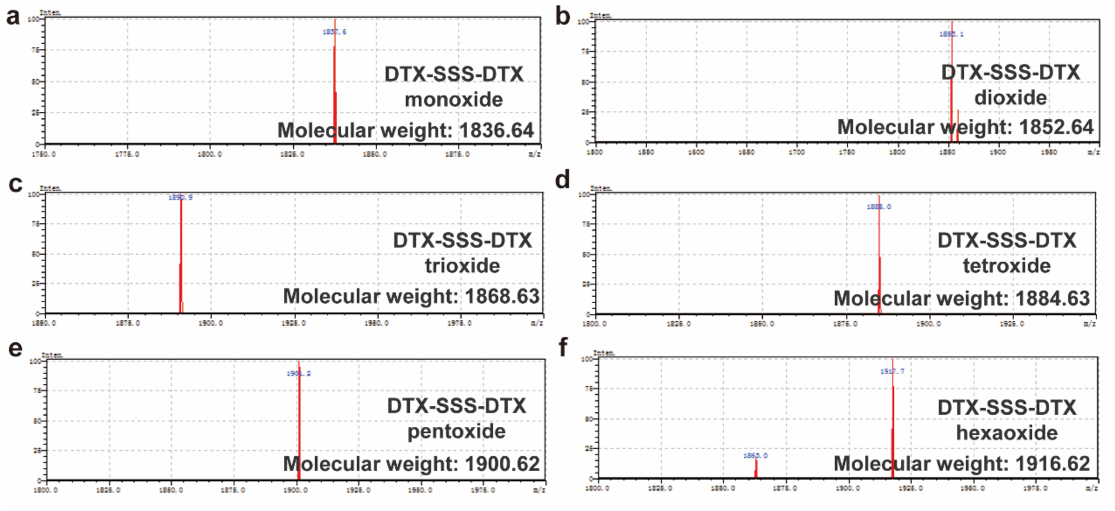

Supplementary Figure 23. Mass spectra of intracellular oxidation-responsive intermediates of DTX-SSS-DTX NPs. **a-f** monoxide to hexaoxide of DTX-SSS-DTX.

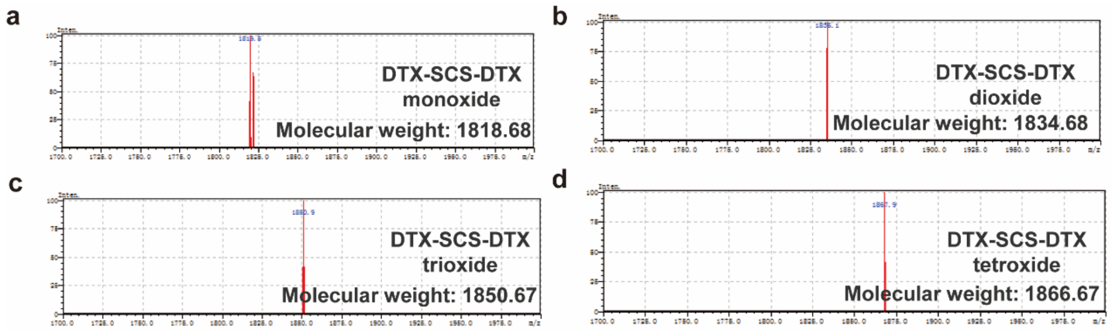

Supplementary Figure 24. Mass spectra of intracellular oxidation-responsive intermediates of DTX-SCS-DTX NPs. **a-d** monoxide to tetroxide of DTX-SCS-DTX.

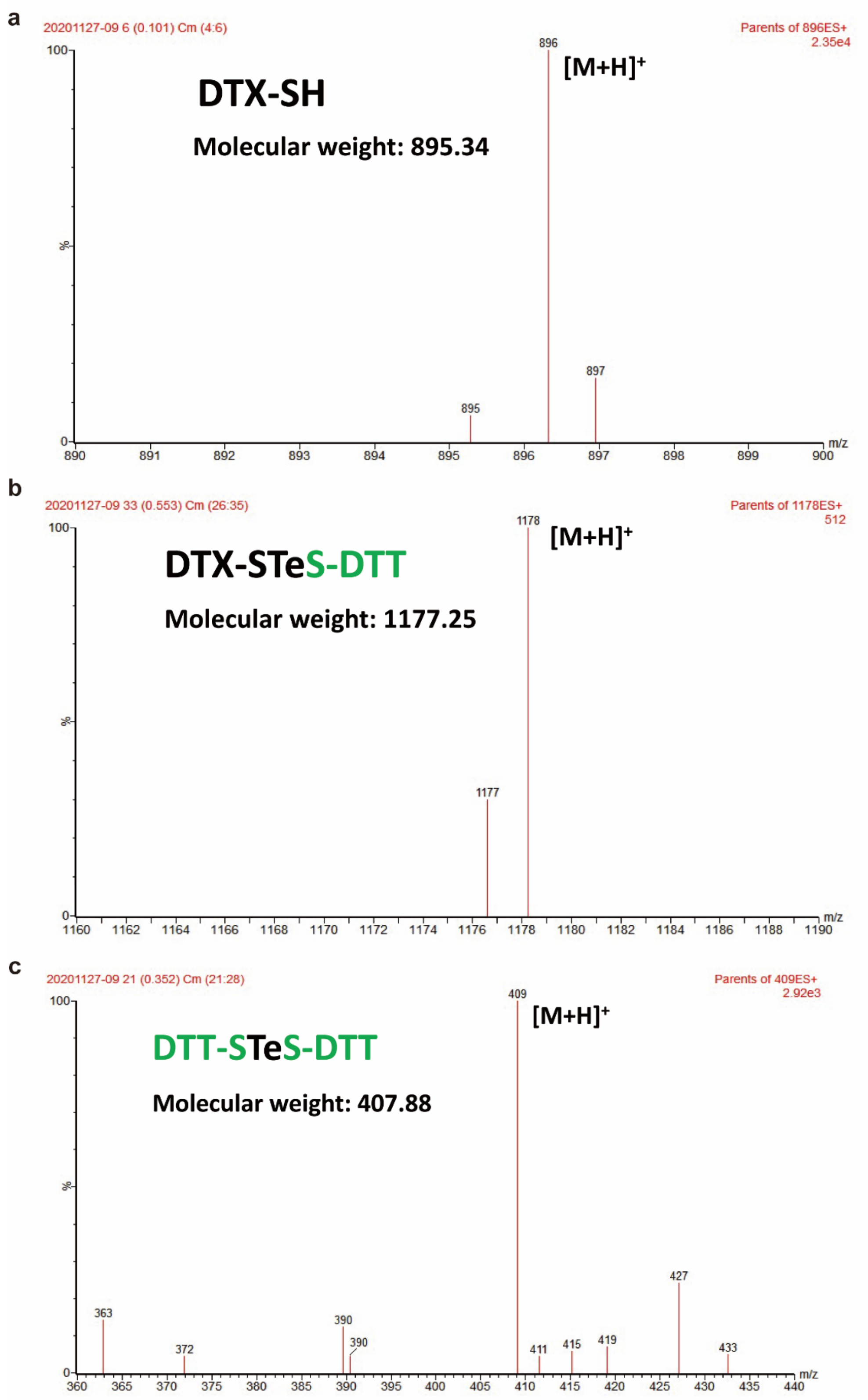

Supplementary Figure 25. Mass spectra of DTX-STeS-DTX after incubated with DTT containing release media. **a** DTX-SH. **b** DTX-STeS-DTT. **c** DTT-STeS-DTT.

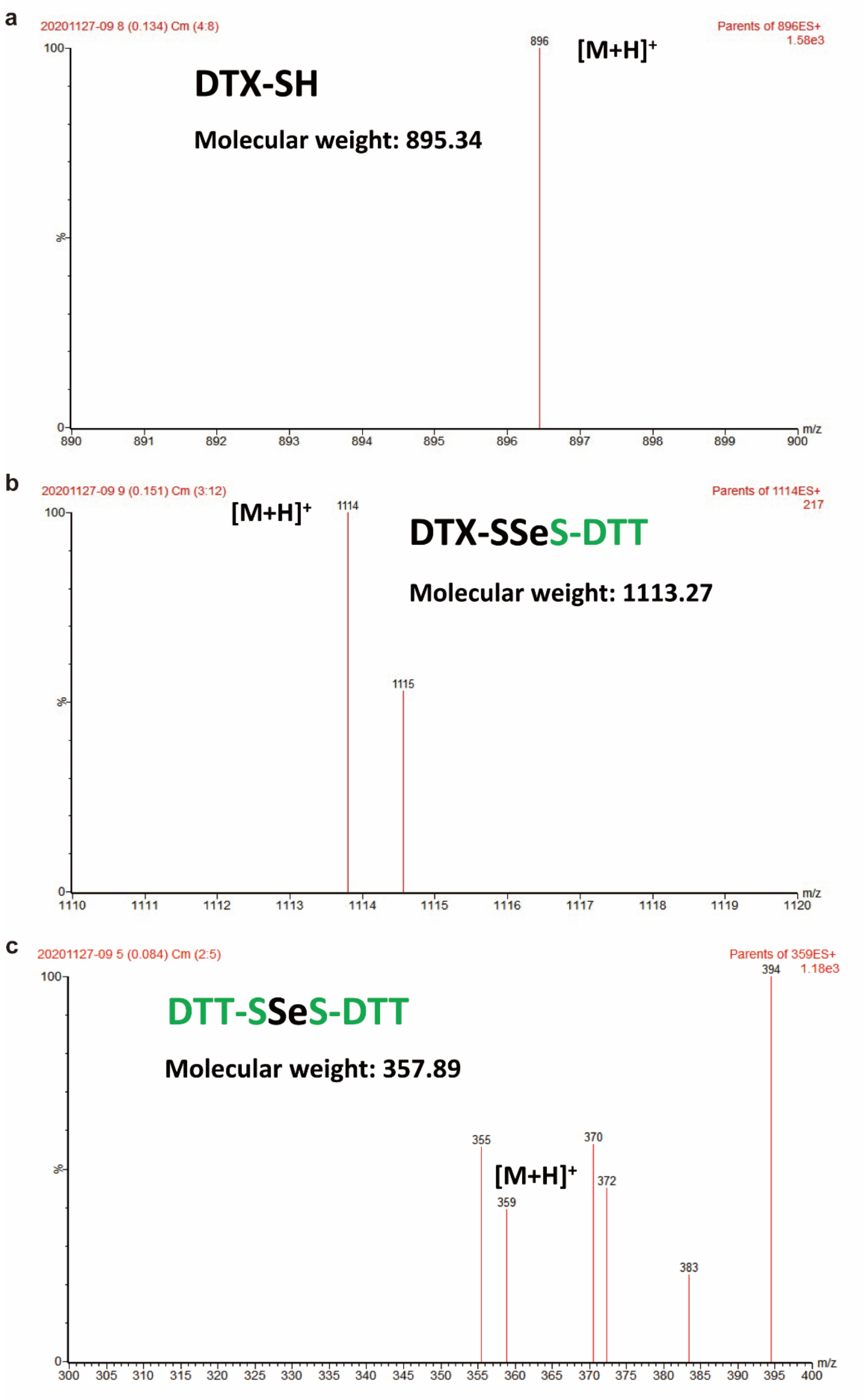

Supplementary Figure 26. Mass spectra of DTX-SSeS-DTX after incubated with DTT containing release media. **a** DTX-SH. **b** DTX-SSeS-DTT. **c** DTT-SSeS-DTT.

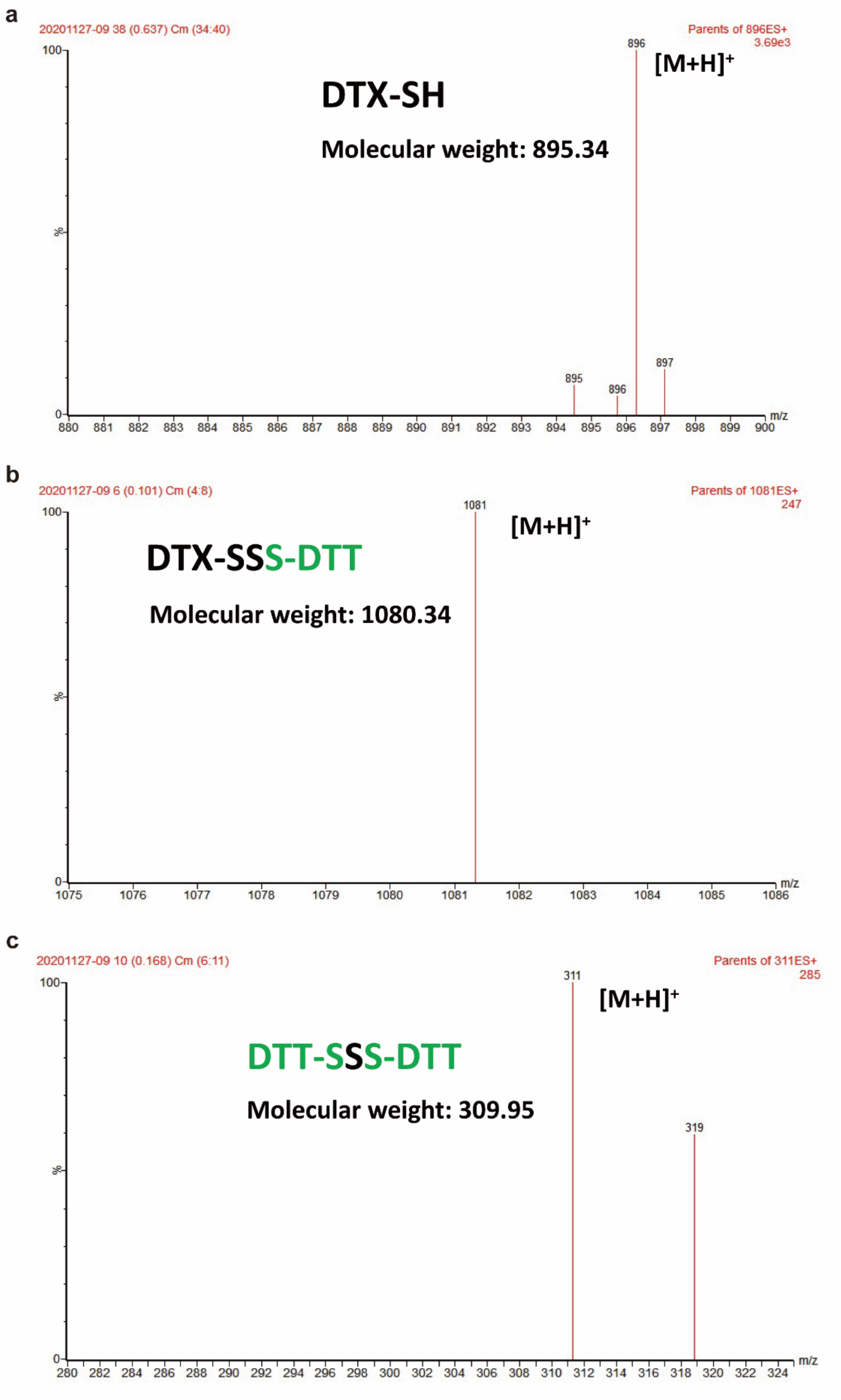

Supplementary Figure 27. Mass spectra of DTX-SSS-DTX after incubated with DTT containing release media. **a** DTX-SH. **b** DTX-SSS-DTT. **c** DTT-SSS-DTT.

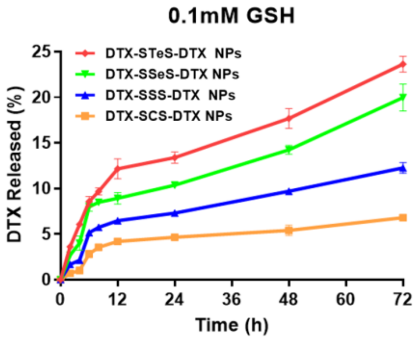

Supplementary Figure 28. In vitro reduction-responsive drug release of HPNAs in the presence of 0.1 mM GSH. Data are presented as mean ± SD (n = 3 independent experiments). Source data are provided as a Source Data file.

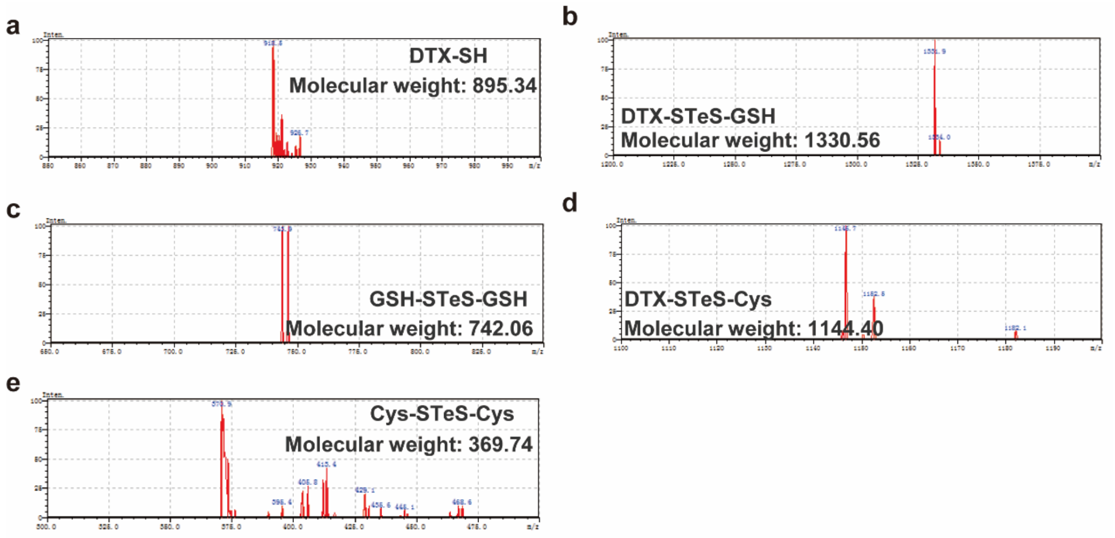

Supplementary Figure 29. Mass spectra of intracellular reduction-responsive intermediates of DTX-STeS-DTX NPs. **a** DTX-SH. **b** DTX-STeS-GSH. **c** GSH-STeS-GSH. **d** DTX-STeS-Cys. **e** Cys-STeS-Cys.

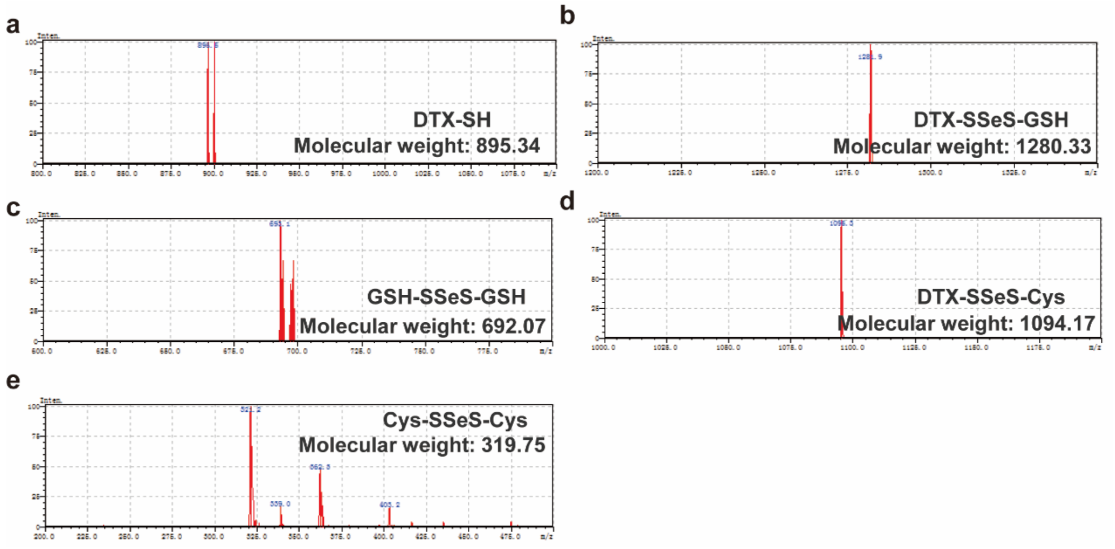

Supplementary Figure 30. Mass spectra of intracellular reduction-responsive intermediates of DTX-SSeS-DTX NPs. **a** DTX-SH. **b** DTX-SSeS-GSH. **c** GSH-SSeS-GSH. **d** DTX-SSeS- Cys. **e** Cys-SSeS-Cys.

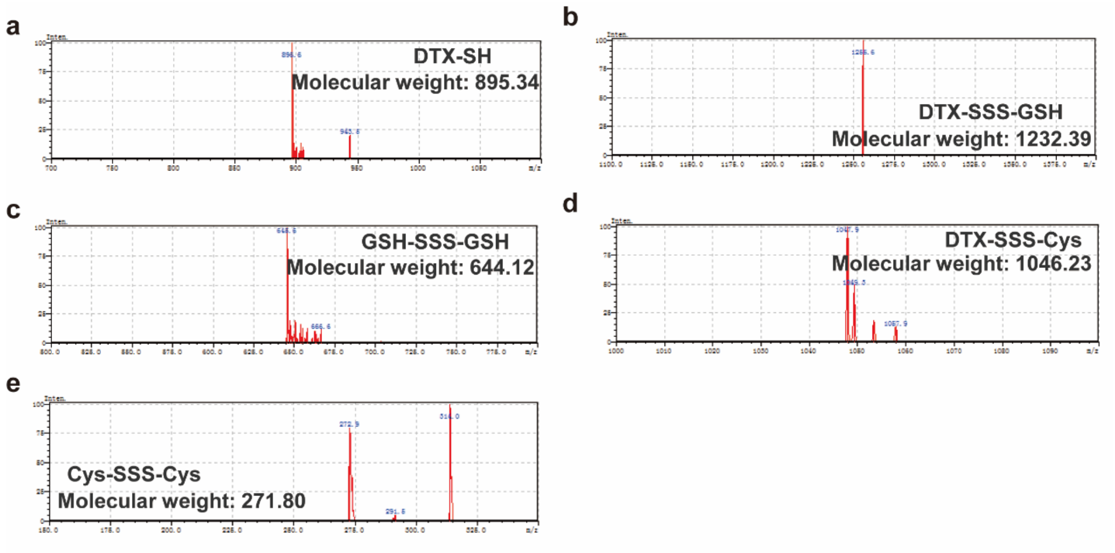

Supplementary Figure 31. Mass spectra of intracellular reduction-responsive intermediates of DTX-SSS-DTX NPs. **a** DTX-SH. **b** DTX-SSS-GSH. **c** GSH-SSS-GSH. **d** DTX-SSS-Cys. **e** Cys-SSS-Cys.

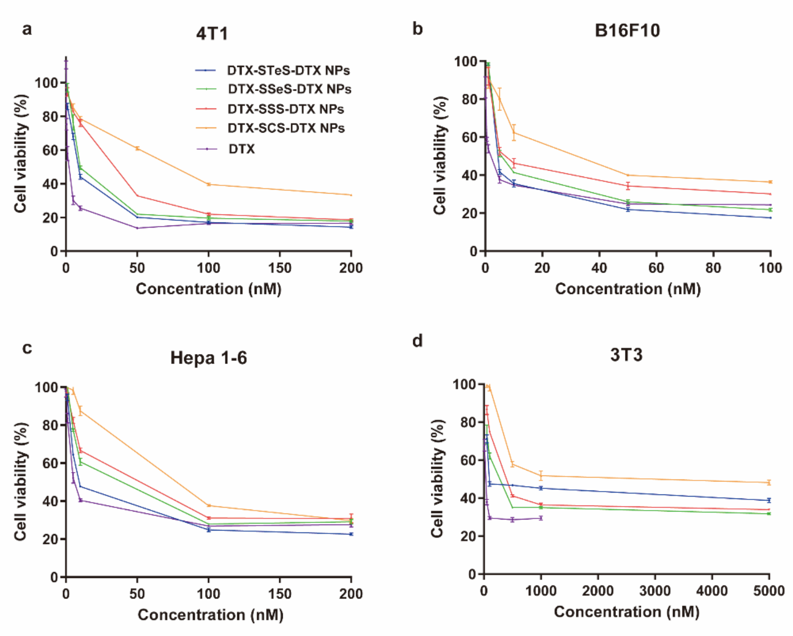

Supplementary Figure 32. Cell viability treated with various concentrations of Taxol and HPNAs. **a** 4T1 cells, **b** B16F10 cells, **c** Hepa 1-6 cells, and **d** 3T3 cells. All Data are presented as mean ± SD (n = 3 independent experiments). Source data are provided as a Source Data file.

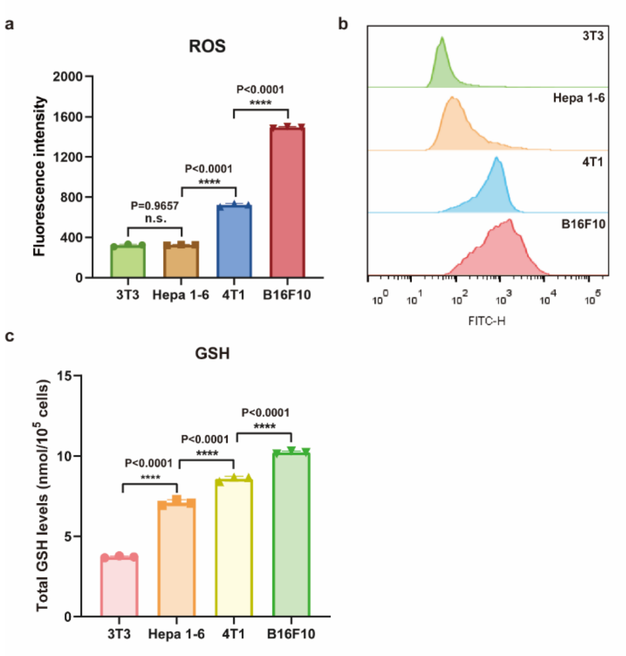

Supplementary Figure 33. Intracellular ROS and GSH levels of the 3T3, Hepa 1-6, 4T1, and B16F10 cells. **a** Flow cytometry analysis for intracellular ROS of the different cells. Data are presented as mean ± SD (n = 3 independent experiments). One-way ANOVA with Tukey's multiple comparisons test was used for the analysis of data and adjusted P value. It was one-sided. Source data are provided as a Source Data file. **b** Histogram of flow analysis for intracellular ROS. **c** Intracellular GSH concentrations of the 3T3, Hepa 1-6, 4T1, and B16F10 cells. Data are presented as mean ± SD (n = 3 independent experiments). One-way ANOVA with Tukey's multiple comparisons test was used for the analysis of data and adjusted P value. It was one-sided. Source data are provided as a Source Data file.

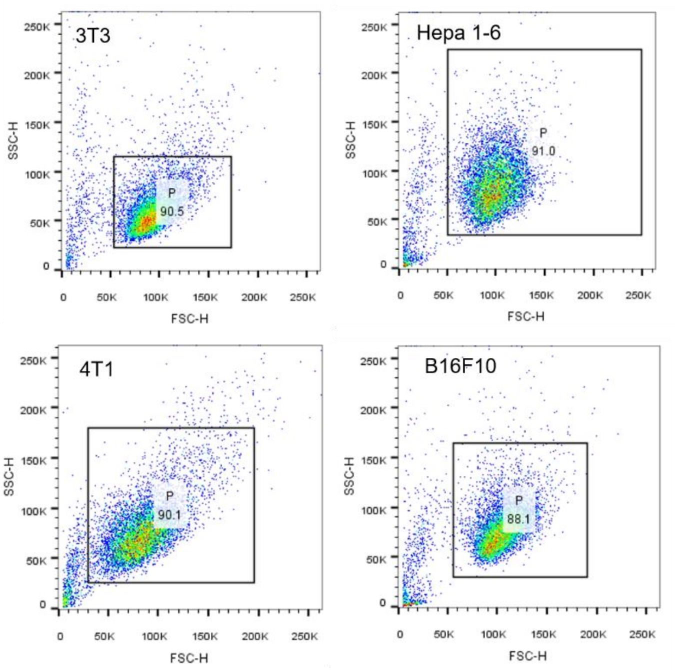

Supplementary Figure 34. The gating strategy for intracellular ROS levels of the 3T3, Hepa 1-6, 4T1, and B16F10 cells.

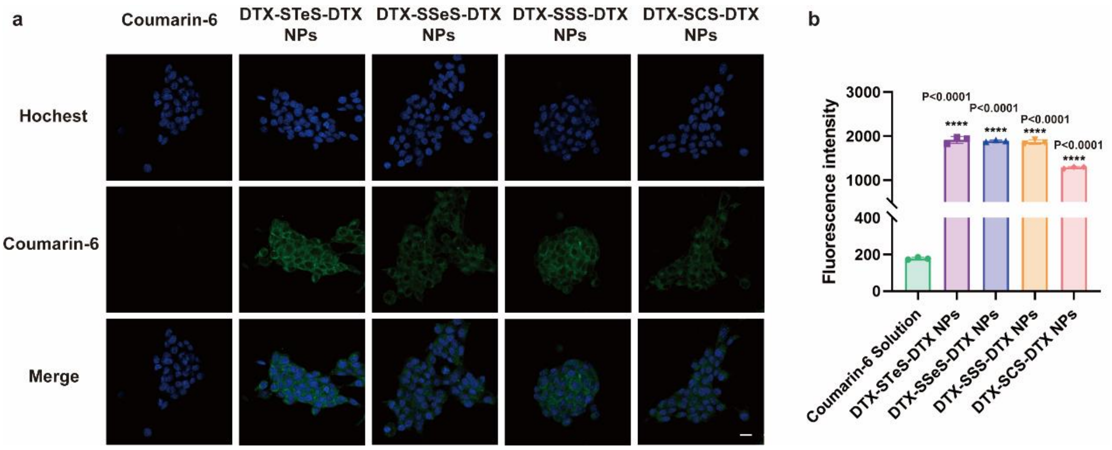

Supplementary Figure 35. Cellular uptake of HPNAs at 0.5 h. **a** Cellular uptake of free coumarin-6 or coumarin-6-labeled HPNAs at 0.5 h. Scale bar represents 10 μm. Experiment was repeated twice independently with similar results. **b** Results of cell uptake by flow cytometry. Data are presented as mean ± SD (n = 3 independent experiments). One-way ANOVA (one-sided) with Dunnett's multiple comparisons test was used for the analysis of data and adjusted P value. Source data are provided as a Source Data file.

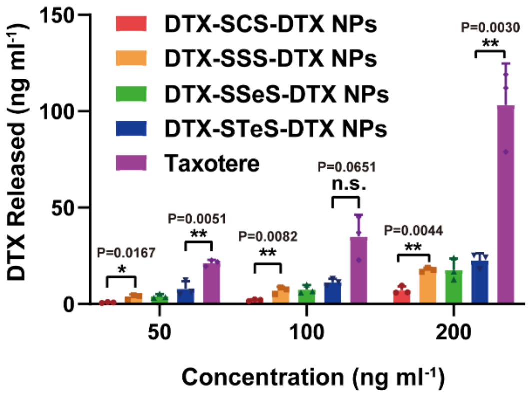

Supplementary Figure 36. Free DTX released from HPNAs after incubation with 4T1 cells for 48 h. Data are presented as mean ± SD (n = 3 independent experiments). n.s (no significance) P > 0.05, * P < 0.05 and ** P < 0.01 by two-tailed Student's t-test. Source data are provided as a Source Data file.

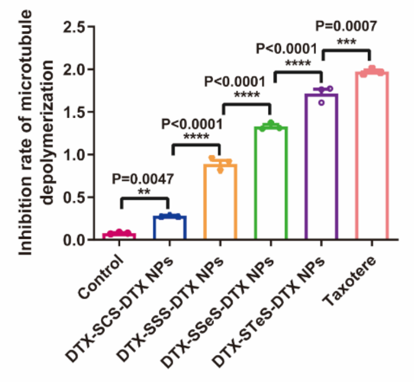

Supplementary Figure 37. The fluorescence intensity of inhibition rate of microtubule depolymerization in CLSM images analyzed by Image J. Data are presented as mean ± SD (n = 3 independent experiments). One-way ANOVA with Tukey's multiple comparisons test was used for the analysis of data and adjusted P value. It was one-sided. ** $P < 0.01$, ***$P < 0.001$ and ****$P < 0.0001$. Source data are provided as a Source Data file.

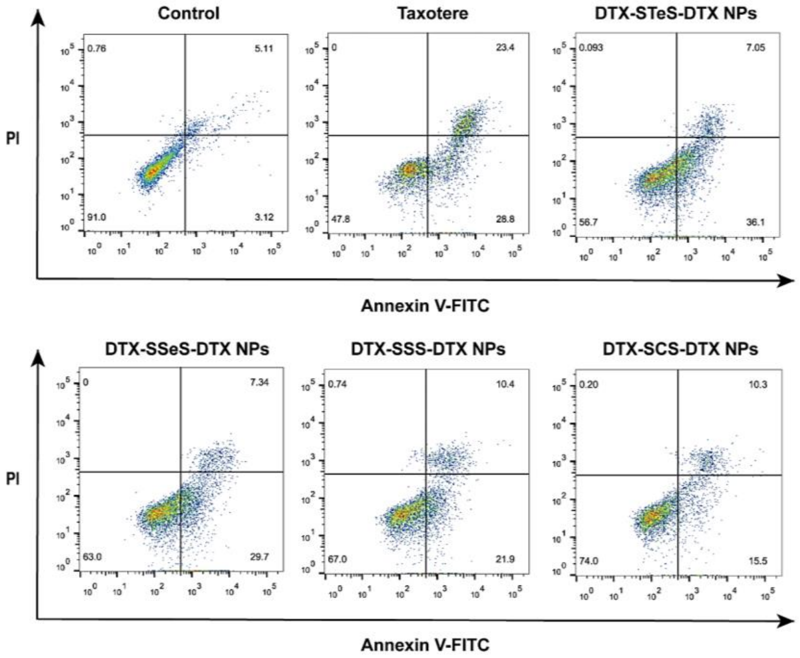

Supplementary Figure 38. Cellular apoptosis assay of Taxotere and HPNAs in 4T1 cells.

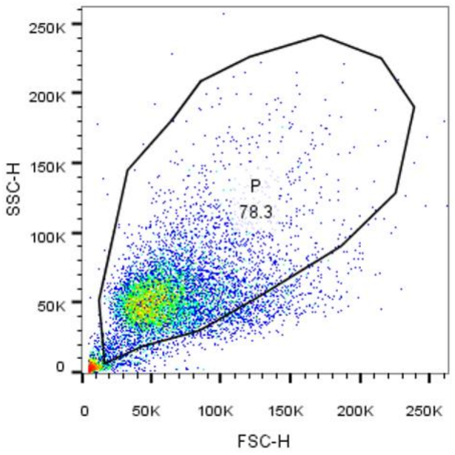

Supplementary Figure 39. The gating strategy for cellular apoptosis assay in 4T1 cells.

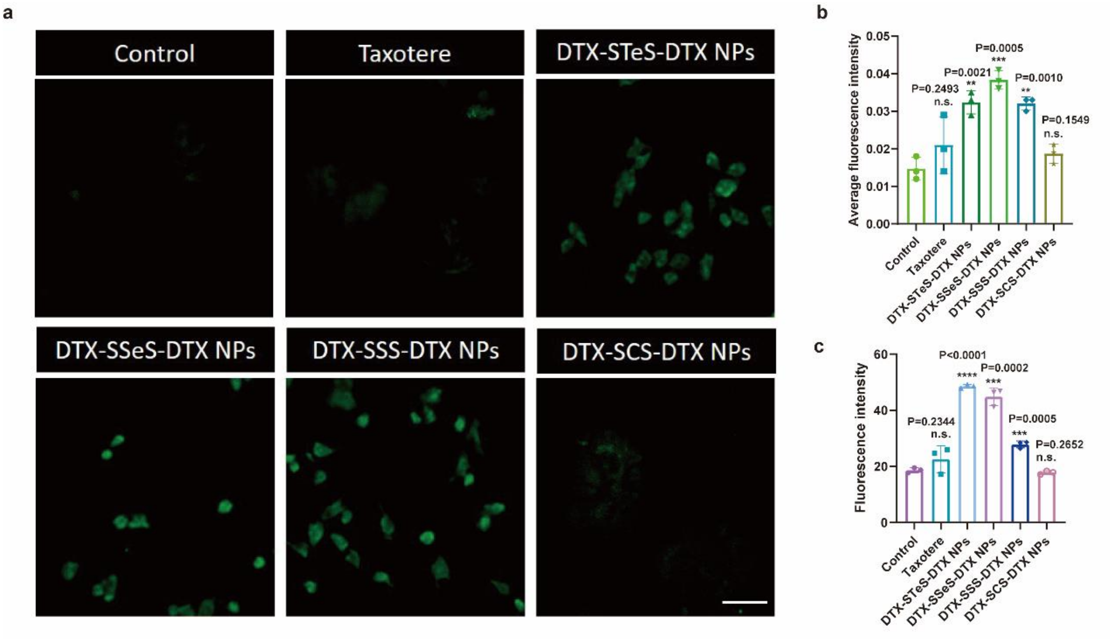

Supplementary Figure 40. Intracellular ROS levels of the 4T1 after treated with Taxotere or HPNAs. **a** CLSM images. Scale bar represents 20 μm. Experiment was repeated twice independently with similar results. **b** Fluorescence quantitative results of CLSM images. Data are presented as mean ± SD (n = 3 independent experiments). Source data are provided as a Source Data file. **c** Flow cytometry analysis for intracellular ROS. Data are presented as mean ± SD (n = 3 independent experiments). n.s (no significance) P > 0.05, ** P < 0.01, *** P < 0.001, and **** P < 0.0001 by two-tailed Student's t test. Source data are provided as a Source Data file.

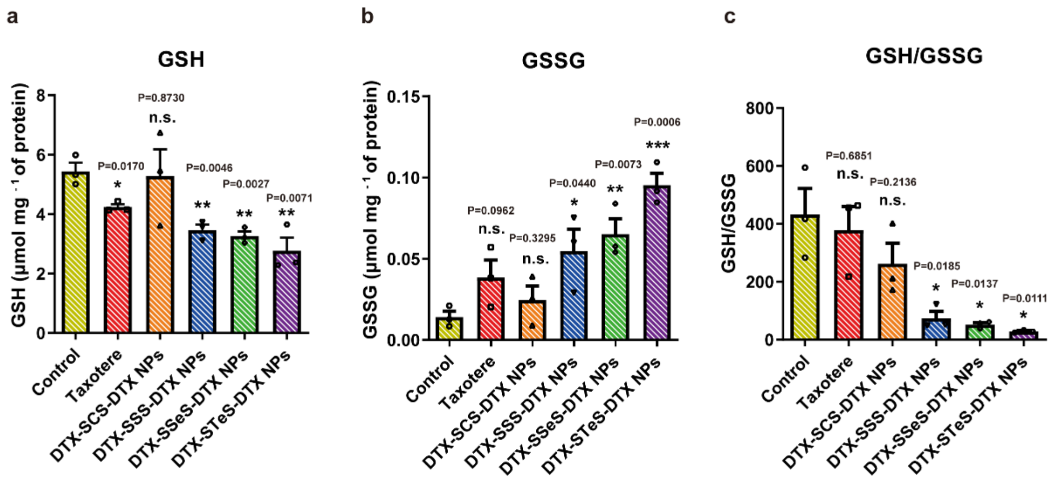

Supplementary Figure 41. Flow cytometric analysis for intracellular GSH/GSSG levels of the 4T1 after treated with Taxotere or HPNAs. **a** The concentration of GSH. **b** The concentration of GSSG. **c** The concentration ratio of GSH and GSSG. All data are presented as mean ± SD (n = 3 independent experiments). n.s (no significance) P > 0.05, * P < 0.05, ** P < 0.01, and *** P < 0.001 by two-tailed Student's t test. Source data are provided as a Source Data file.

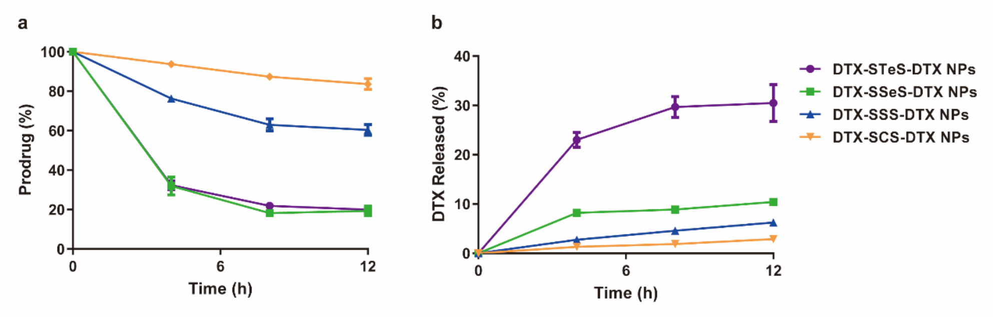

Supplementary Figure 42. The chemical stability of HPNAs in the fresh rat plasma. **a** Degradation of prodrugs. **b** The released DTX from HPNAs. Data are presented as mean ± SD (n = 3 independent experiments). Source data are provided as a Source Data file.

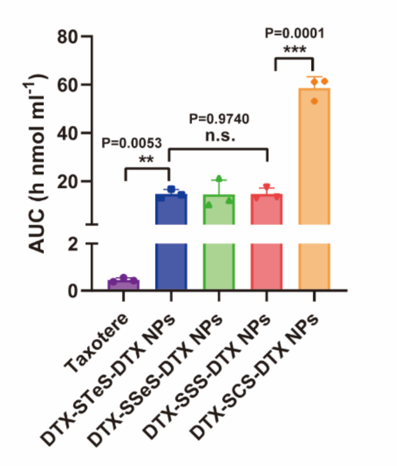

Supplementary Figure 43. Area under curve (AUC) of Taxotere and HPNAs. Data are presented as mean ± SD (n = 3 rats). n.s (no significance) P > 0.05, ** P < 0.01 and ***P < 0.001 by two-tailed Student's t-test. Source data are provided as a Source Data file.

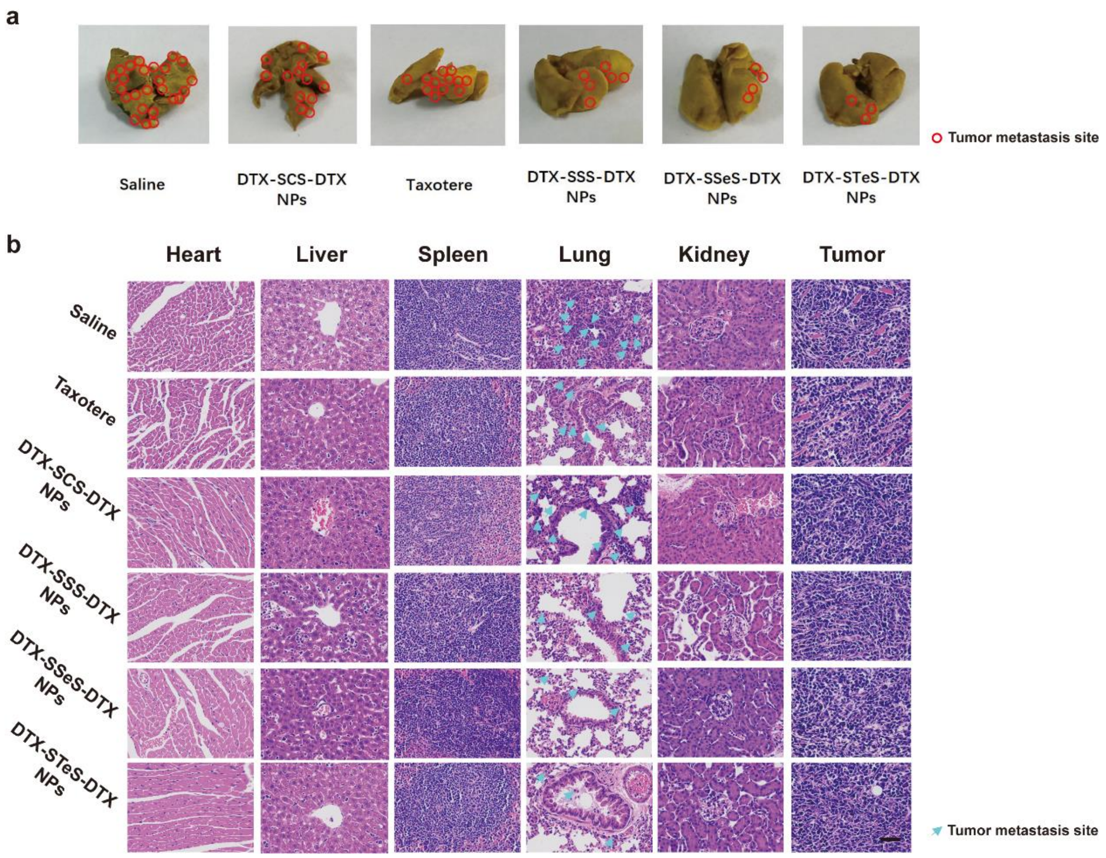

Supplementary Figure 44. Lung metastasis and H&E staining at doses of 2 mg kg^{-1}. **a** Lung metastasis of 4T1 tumor-bearing BALB/c mice after treatments. The red circles represent the location of tumor metastasis. **b** H&E staining. The major organs and tumor sections of 4T1 tumor-bearing BALB/c mice were prepared after the last treatments. The blue arrows represent the location of tumor metastasis. Scale bar represents 100 µm. Experiment was repeated three times independently with similar results.

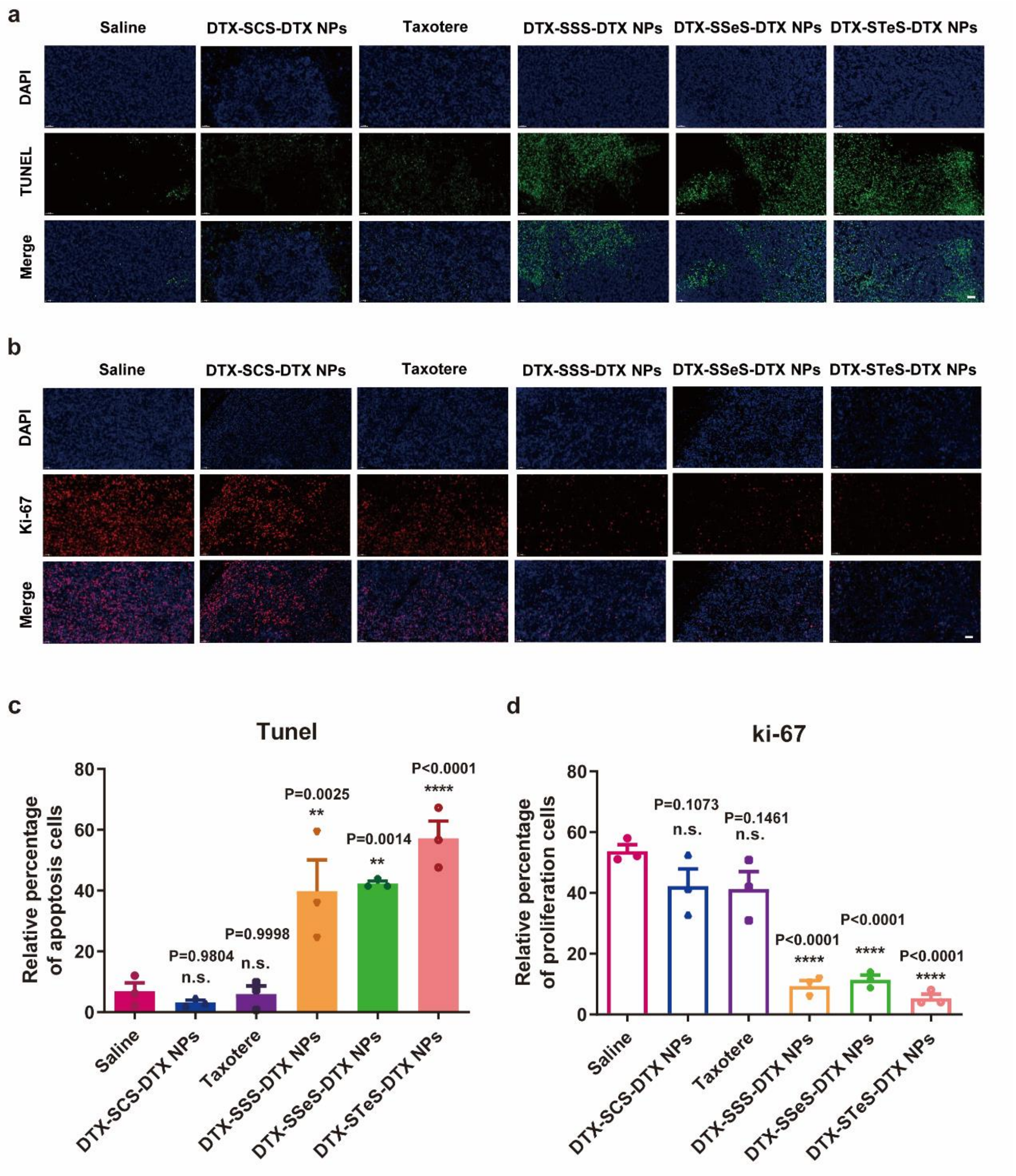

Supplementary Figure 45. TUNEL and Ki67 assay of 4T1 tumor sections at doses of 2 mg kg^{-1} after the last treatment. **a-b** Fluorescence microscope image. Scale bar represents 50 μm. **c-d** Fluorescence quantitative results of TUNEL and Ki67 assay. Data are presented as mean ± SD (n = 3 mice). One-way ANOVA with Dunnett's multiple comparisons test was used for the analysis of data and adjusted P value. It was one-sided. Source data are provided as a Source Data file.

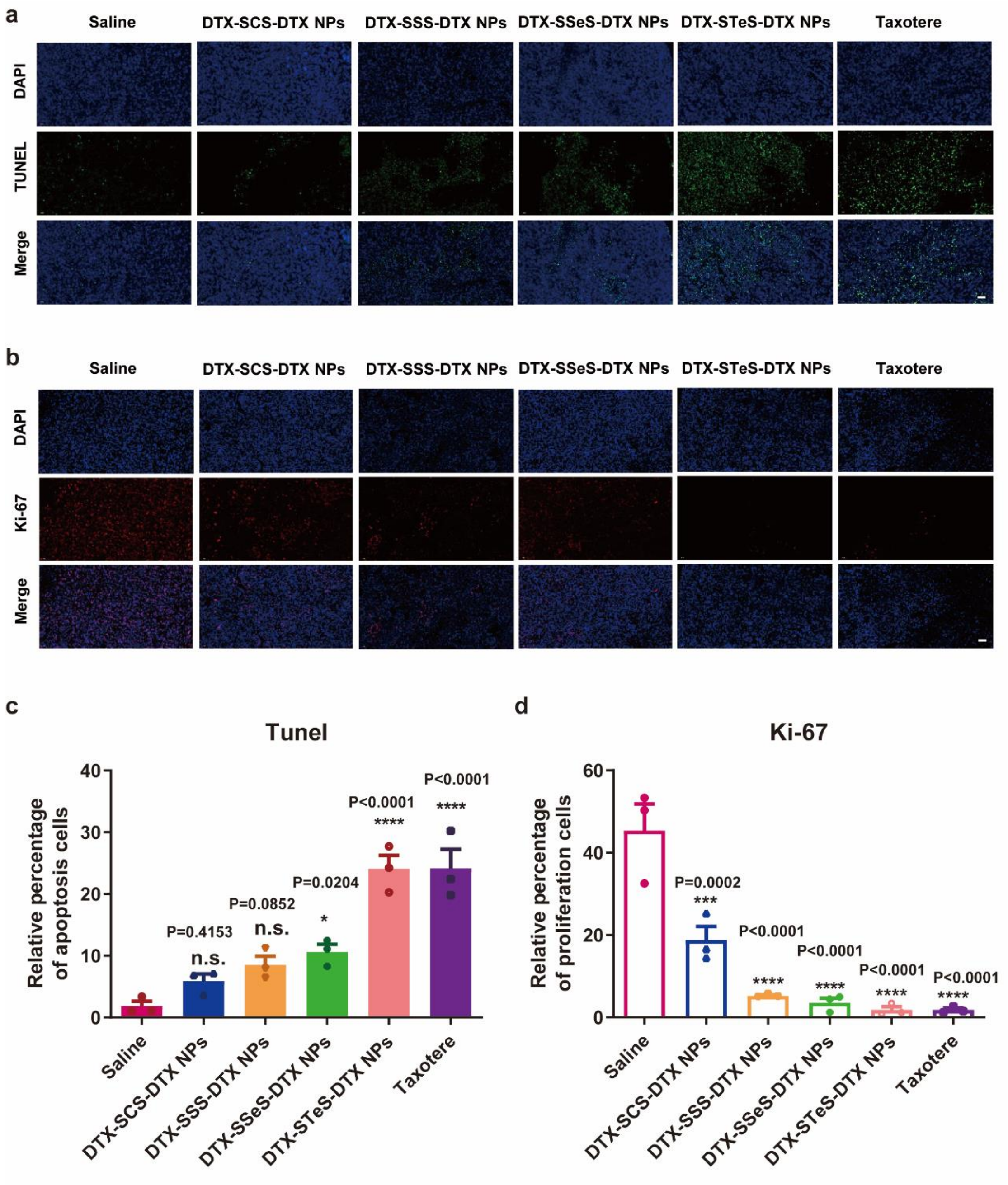

Supplementary Figure 46. TUNEL and Ki67 assay of 4T1 tumor sections at doses of 10 mg kg^{-1} after the last treatment. **a**-**b** Fluorescence microscope image. Scale bar represents 50 μm. **c**-**d** Fluorescence quantitative results of TUNEL and Ki67 assay. Data are presented as mean ± SD (n = 3 mice). One-way ANOVA with Dunnett's multiple comparisons test was used for the analysis of data and adjusted P value. It was one-sided. Source data are provided as a Source Data file.

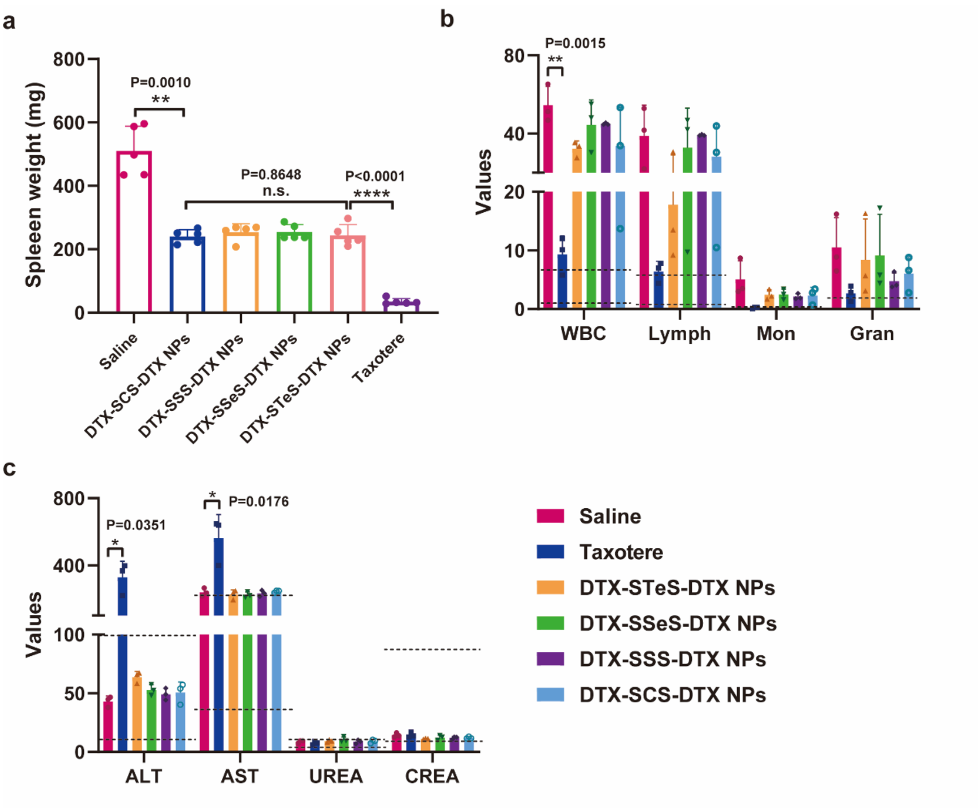

Supplementary Figure 47. Safety analysis of HPNAs at a dose of 10 mg kg^{-1}. **a** Spleen weight of 4T1 tumor-bearing BALB/c mice after treatments. Data are presented as mean ± SD (n = 5 mice). Source data are provided as a Source Data file. **b** Blood count. WBC: white blood cell count (10^9 L^{-1}), Lymph: lymphocyte count (10^9 L^{-1}), Mon: monocyte count (10^9 L^{-1}), Gran: granulocyte count (10^9 L^{-1}). Data are presented as mean ± SD (n = 3 mice). Source data are provided as a Source Data file. **c** Hepatorenal function parameters. AST: aspartate aminotransferase (U L^{-1}); ALT: alanine aminotransferase (U L^{-1}), BUN: blood urea nitrogen (mmol L^{-1}), CREA: creatinine (μmol L^{-1}). The dotted line represents the reference value of normal mice. Data are presented as mean ± SD (n = 3 mice). n.s (no significance) P > 0.05, * P < 0.05, ** P < 0.01, *** P < 0.001, and **** P < 0.0001 by two-tailed Student's t test. Source data are provided as a Source Data file.

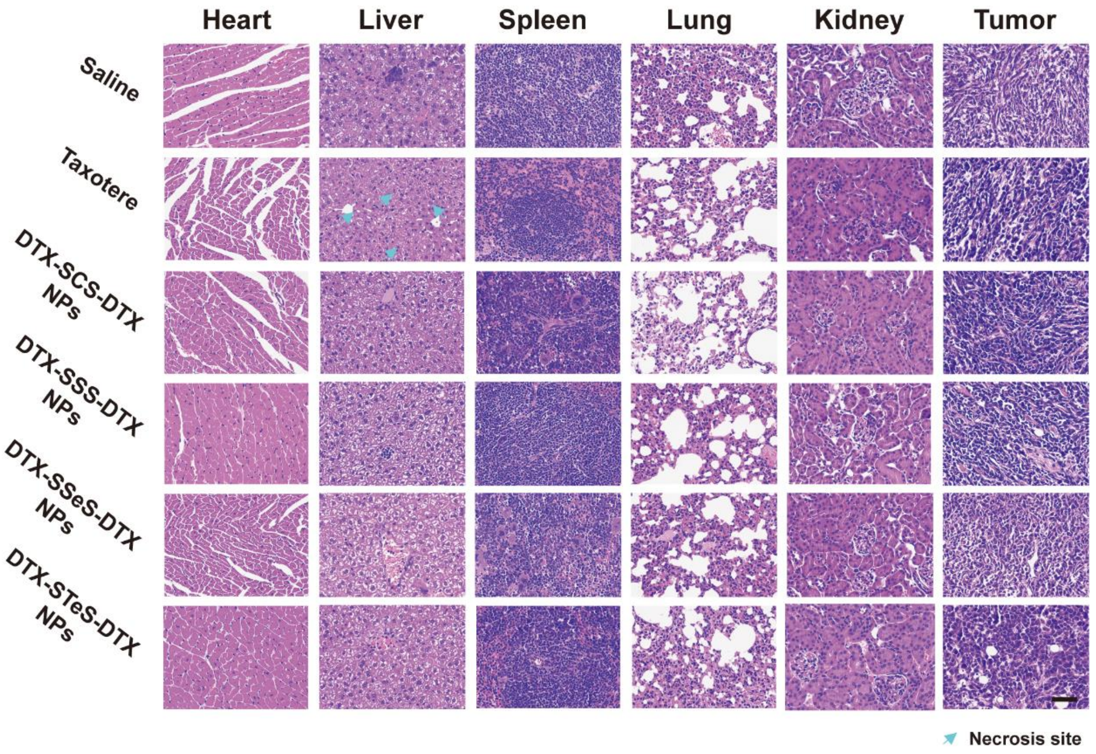

Supplementary Figure 48. H&E staining at doses of 10 mg kg^{-1}. The major organs and tumor sections of 4T1 tumor-bearing BALB/c mice were prepared after the last treatments. The blue arrows represent the location of necrotic site. Scale bar represents 100 µm. Experiment was repeated three times independently with similar results.

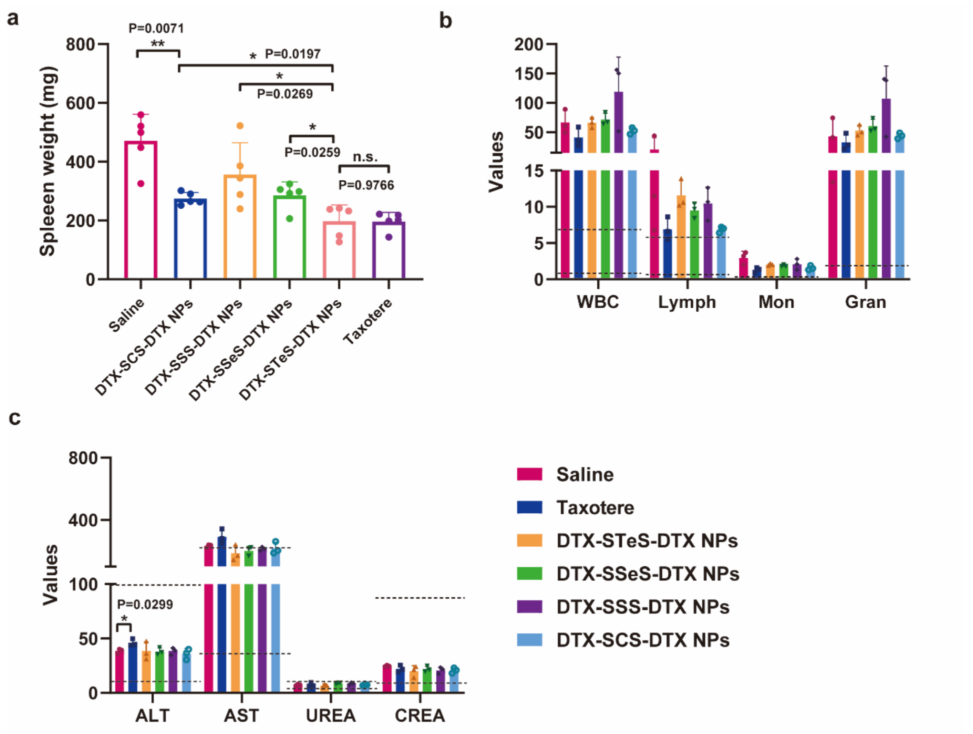

Supplementary Figure 49. Safety analysis of HPNAs at a dose of 2 mg kg^{-1}. **a** Spleen weight. Data are presented as mean ± SD (n = 5 mice). Source data are provided as a Source Data file. **b** Blood count. WBC: white blood cell count (10^9 L^{-1}), Lymph: lymphocyte count (10^9 L^{-1}), Mon: monocyte count (10^9 L^{-1}), Gran: granulocyte count (10^9 L^{-1}). Data are presented as mean ± SD (n = 3 mice). Source data are provided as a Source Data file. **c** Hepatorenal function parameters. AST: aspartate aminotransferase (U L^{-1}); ALT: alanine aminotransferase (U L^{-1}), BUN: blood urea nitrogen (mmol L^{-1}), CREA: creatinine (μmol L^{-1}). The dotted line represents the reference value of normal mice. Data are presented as mean ± SD (n = 3 mice). n.s (no significance) P > 0.05, * P < 0.05, ** P < 0.01, *** P < 0.001, and **** P < 0.0001 by two-tailed Student's t test. Source data are provided as a Source Data file.

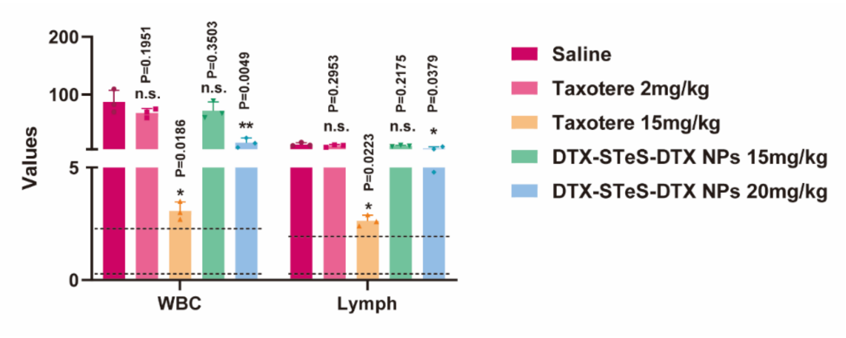

Supplementary Figure 50. Blood count of 4T1 tumor-bearing BALB/c mice treated with HPNAs at the high doses. WBC: white blood cell count (10^9 L^{-1}), Lymph: lymphocyte count (10^9 L^{-1}). The dotted line represents the reference value of normal mice. Data are presented as mean ± SD (n = 3 mice). n.s (no significance) $P > 0.05$, * $P < 0.05$ and ** $P < 0.01$ by two-tailed Student's t test. Source data are provided as a Source Data file.

Supplementary Tables

Supplementary Table 1. Characterization of PEGylated HPNAs.

Nanoassemblies	Size (nm)	PDI	Zeta (mV)	DL (w/w, %)
DTX-STeS-DTX NPs	96	0.15	-24.3	67.38
DTX-SSeS-DTX NPs	95	0.19	-21.0	69.18
DTX-SSS-DTX NPs	97	0.14	-19.8	71.00
DTX-SCS-DTX NPs	109	0.12	-20.3	71.70

Supplementary Table 2. Total binding energy of prodrug and DSPE-PEG_{2K} system.

Contribution (kJ mol^{-1})	DTX-SCS-DTX	DTX-SSS-DTX	DTX-SSeS-DTX	DTX-STeS-DTX
ΔE_{vdW}	-13032.8	-14887.9	-15736.9	-16832.9
ΔE_{elec}	-1123.1	-1205.2	-1324.7	-1407.8
ΔG_{polar}	5251.0	5846.6	5364.5	5443.9
$\Delta G_{nonpolar}$	-1071.2	-1225.7	-1351.7	-1458.9
ΔG_{total}	-9976.1	-11472.2	-13048.8	-14255.7

Total binding energy（Δ_{Gtotal}）= Van der Waals（Δ_{EvdW}）+ Electrostatic energy（Δ_{Eelec}）+ Polarization solvation energy（Δ_{Gpolar}）+ Nonpolarized solvation energy（$\Delta_{Gnonpolar}$）

Supplementary Table 3. IC_{50} values (nM) of Taxol and HPNAs against three tumor cell lines and one normal cell line.

Cell lines	Taxotere	DTX-STeS-DTX NPs	DTX-SSeS-DTX NPs	DTX-SSS-DTX NPs	DTX-SCS-DTX NPs
4T1	2.11±0.24	10.19±0.66	15.55±1.11	29.44±1.92	78.38±3.06
B16-F10	2.28±0.35	6.83±0.29	10.80±0.61	16.17±4.79	43.94±12.17
Hepa 1-6	11.11±1.67	18.45±1.52	37.55±2.80	54.81±7.23	83.84±4.91
3T3	28.39±7.34	392.50±102.07	378.67±86.52	1007.13±26.55	3520.00±699.88

Data are presented as mean ± SD (n = 3 independent experiments).

Supplementary Table 4. The selectivity index (SI) of Taxotere and HPNAs between normal cells and tumor cells.

Cell lines	Taxotere	DTX-STeS-DTX NPs	DTX-SSeS-DTX NPs	DTX-SSS-DTX NPs	DTX-SCS-DTX NPs
4T1	11.85	32.78	26.71	35.70	43.79
B16-F10	12.15	57.30	38.07	64.37	78.14
Hepa 1-6	2.52	21.29	10.97	23.88	40.86

SI=($IC_{50normal}$)/($IC_{50tumor}$). ("$IC_{50normal}$ and $IC_{50tumor}$" represent the IC_{50} of HPNAs toward normal cells and tumor cells).

Supplementary Table 5. Pharmacokinetic parameters of Taxotere and HPNAs.

Formulations	Drug[a)]	AUC_{0-24h}[b)]	CL[c)]	MRT_{0-24h}[d)]
Taxotere	DTX	0.46±0.09	8.74±1.69	2.31±0.70
DTX-STeS-DTX NPs	DTX-STeS-DTX	14.71±1.82	0.27±0.03	0.10±0.01
	DTX	2.54±0.12	1.50±0.13	2.20±0.40
DTX-SSeS-DTX NPs	DTX-SSeS-DTX	14.54±5.90	0.30±0.10	0.48±0.05
	DTX	2.26±0.17	1.71±0.20	2.95±0.29
DTX-SSS-DTX NPs	DTX-SSS-DTX	14.65±2.48	0.28±0.05	0.39±0.03
	DTX	0.71±0.06	5.01±0.55	5.63±0.81
DTX-SCS-DTX NPs	DTX-SCS-DTX	58.58±4.68	0.07±0.00	2.08±0.73
	DTX	0.18±0.03	5.09±1.41	11.34±0.2

a) Prodrugs and the released DTX were measured by LC-MS. b) Area under the plasma concentration-time curve (nmol h mL^{-1}). c) Plasma clearance (mL h^{-1} kg^{-1}). d) Mean retention time (h). Data are presented as mean ± SD (n = 3 mice).
